# The TAS1R2 sweet taste receptor regulates skeletal muscle mass and fitness

**DOI:** 10.21203/rs.3.rs-2475555/v1

**Published:** 2023-02-09

**Authors:** Joan Serrano, Jordan Boyd, Carter Mason, Kathleen R Smith, Katalin Karolyi, Saki Kondo, Ian S Brown, Santosh K Maurya, Nishita N Meshram, Vanida Serna, Joshua Gilger, Daniel A Branch, Stephen J Gardell, Kedryn K Baskin, Julio E Ayala, Richard E Pratley, Bret H Goodpaster, Paul M Coen, George A Kyriazis

**Affiliations:** 1 Biological Chemistry & Pharmacology, College of Medicine, The Ohio State University, Columbus, OH, USA; 2 Physiology and Cell Biology, College of Medicine, The Ohio State University, Columbus, OH, USA; 3 Translational Research Institute, Advent Health, Orlando, FL, USA; 4 Molecular Physiology & Biophysics, Vanderbilt University School of Medicine, Nashville, TN, USA

## Abstract

Muscle fitness and mass deteriorate under the conditions of obesity and aging for reasons yet to be fully elucidated. Herein, we describe a novel pathway linking peripheral nutrient sensing and skeletal muscle function through the sweet taste receptor TAS1R2 and the involvement of ERK2-PARP1-NAD signaling axis. Muscle-specific deletion of TAS1R2 (mKO) in mice produced elevated NAD levels due to suppressed PARP1 activity, improved mitochondrial function, increased muscle mass and strength, and prolonged running endurance. Deletion of TAS1R2 in obese or aged mice also ameliorated the decline in muscle mass and fitness arising from these conditions. Remarkably, partial loss-of-function of TAS1R2 (rs35874116) in older, obese humans recapitulated the healthier muscle phenotype displayed by mKO mice in response to exercise training. Our findings show that inhibition of the TAS1R2 signaling in skeletal muscle is a promising therapeutic approach to preserve muscle mass and function.

Skeletal muscle function and mass can adapt to environmental stimuli by integrating mechanical, hormonal, neuronal, and metabolic pathways. This process involves the activation of sensory pathways to meet energy demands and balance anabolic and catabolic mechanisms. However, the deterioration of these adaptive responses due to aging or obesity can lead to the loss of muscle mass and function. Therefore, it is important to identify and target the molecular networks that preserve or restore muscle health in the development of these conditions.

Nicotinamide adenine dinucleotide (**NAD**) is an endogenous metabolite involved in muscle adaptation. It participates in redox reactions and serves as a substrate for poly(ADP-ribose) polymerases (**PARPs**) and NAD-dependent deacetylases (sirtuins; **SIRT**)^1^. Specifically, the activation of the NAD-SIRT1 axis is crucial for maintaining metabolic health^2^. Depletion of cellular NAD is associated to insulin resistance, diabetes^3^, skeletal muscle atrophy and dysfunction^4^. Strategies that enhance NAD synthesis (e.g. NAD precursor supplementation^5,6^, exercise^7,8^, caloric restriction^9–11^) or block its consumption (e.g. inhibitors of PARPs^12–14^ or CD38^15,16^) can improve mitochondrial function, metabolic flexibility, insulin sensitivity and increase muscle mass. For instance, the direct inhibition of PARP1 increases NAD availability for SIRT1 activation, resulting in improvements in muscle fitness^13,14^. However, the physiological pathways that link energy metabolism to PARP1 activity for the coordination of NAD bioavailability have yet to be established.

G protein-coupled receptors (GPCRs) are central mediators of cellular function that integrate extracellular and intracellular signaling pathways. They are typically activated by hormones and neurotransmitters, but a growing number of specialized GPCRs have been identified that respond to nutrients or endogenous metabolites^17^. Some of these GPCRs are found on the tongue, intestine, and pancreas where they can directly sense energy substrates such as fatty acids, amino acids, or sugars. For example, sugars are sensed by sweet taste receptors (**STR**), which are formed by the obligate heterodimerization of TAS1R2 and TAS1R3, to trigger the secretion of ATP (taste buds)^18^, incretins (L-cells)^19,20^, or insulin (beta-cells)^21,22^. Therefore, sugars such as glucose, which typically enter cells to be used for energy or for generating regulatory metabolites, can also independently activate signal transduction pathways through cell-surface GPCRs. In support of a role of STR in endocrine physiology, whole-body genetic ablation of TAS1R2 (**bKO**) protected mice from metabolic derangements associated with chronic high-fat diet (**HFD**), such as hyperinsulinemia and liver steatosis^23^. Unexpectedly, bKO mice had higher lean mass in response to HFD^23^, suggesting that the role of STR may extend beyond endocrine physiology. These findings prompted us to investigate whether STR signaling exerts muscle-autonomous effects on the regulation of muscle mass and fitness.

## RESULTS

### TAS1R2 regulates skeletal muscle mass and fitness

**Extended Data Figure 1.**
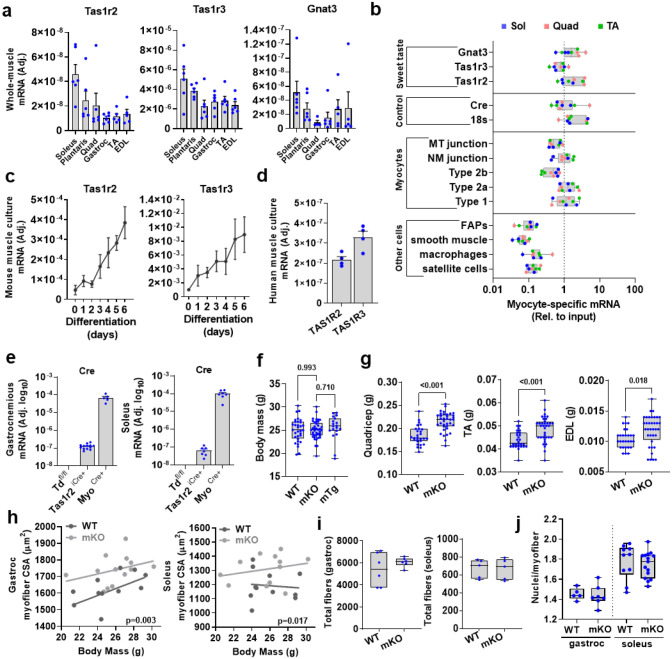
Deletion of TAS1R2 in skeletal muscle induces improvements in skeletal muscle mass. **(a)** Expression of the sweet taste receptor genes, Tas1r2 and Tas1r3, and their coupled Ga, Gnat3, in various skeletal muscles. **(b)** Gene expression in myocytes from soleus (Sol), Quadriceps (Quad) and Tibialis Anterior (TA) relative to whole muscle input control. Myotendinous (MT), neuromuscular (NM), fibro/adipogenic progenitors (FAP). **(c)** Expression of Tas1r2 and Tas1r3 in differentiating mouse myocytes. **(d)** Expression of Tas1r2 and Tas1r3 in differentiated human myocytes. **(e)** Active cre expression in gastrocnemius and soleus of control (Tdfl/fl), Tas1r2-Cre and Myo-Cre mice. **(f)** Body mass in WT, mKO and mTg mice. One-way ANOVA, Sidak post-hoc effect. **(g)** Mass of quadriceps, tibialis anterior (TA) and extensor digitorum longus (EDL) muscles of WT and mKO mice. T-test. **(h)** Simple linear regression between CSA of gastrocnemius (left) or soleus (right) muscles and total body mass of WT and mKO mice. Elevations contrast p value. **(i)** Total number of fibers of gastrocnemius (left) or soleus (right) muscles of WT and mKO mice. T-test. **(j)** Total number of nuclei per myofiber of gastrocnemius or soleus muscles of WT and mKO mice. T-test.

**Figure 1.**
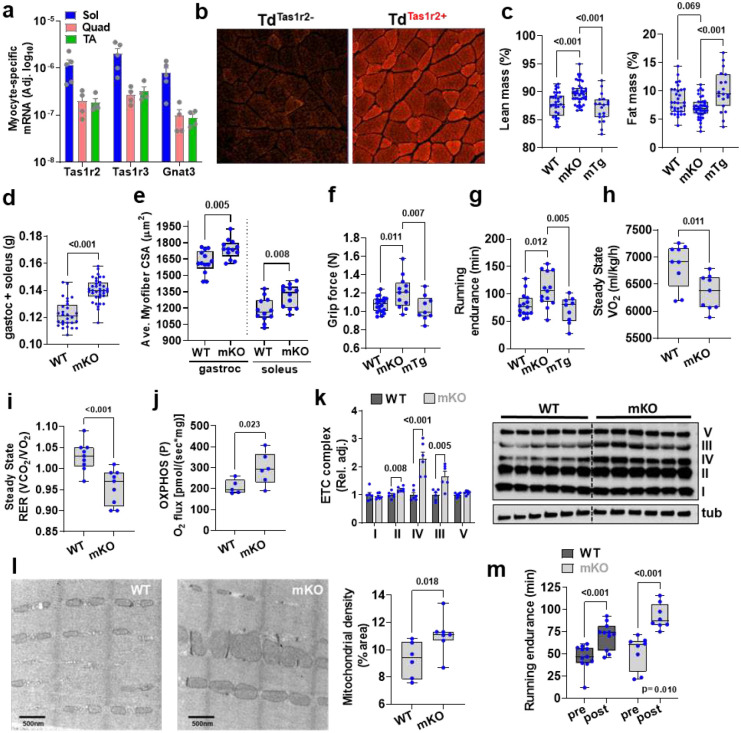
Deletion of TAS1R2 in skeletal muscle induces improvements in skeletal muscle mass and fitness. **(a)** Expression of Tas1r2, Tas1r3 and Gnat3 in myocytes from soleus (Sol), Quadriceps (Quad) and Tibialis Anterior (TA) using RiboTag mRNA pulldown. **(b)** Immunofluorescence of TdTomato in quadriceps of Tas1r2-Cre:TdTomato-fl/fl mice (TdTas1r2+) and negative controls (TdTas1r2−). **(c)** Lean and fat mass in WT, mKO and mTg mice. One-way ANOVA, Sidak post-hoc effect. **(d)** Total mass of gastrocnemius and soleus muscles of WT and mKO mice. T-test. **(e)** Average myofiber cross-sectional area (CSA) of gastrocnemius and soleus muscles of WT and mKO mice. T-test. **(f)** Forelimb grip force of WT, mKO and mTg mice. One-way ANOVA, Sidak post-hoc effect. **(g)** Treadmill running endurance of WT, mKO and mTg mice using constant intensity. One-way ANOVA, Sidak post-hoc effect. **(h)** Steady state oxygen consumption (VO2) of WT and mKO mice during moderate intensity treadmill running. T-test**. (i)** Steady state respiratory exchange ratio (RER) of WT and mKO mice during moderate intensity treadmill running. T-test**. (j)** Respiratory capacity in the ADP-activated state of oxidative phosphorylation (OXPHOS P) of intact muscle fibers from WT and mKO mice. T-test. **(k)** Immunoblotting of mitochondrial electron transport chain (ETC) proteins in WT and mKO muscle. T-test. **(l)** Mitochondrial density and structure of WT and mKO muscle using transmission electron microscopy (TEM). T-test. **(m)** Treadmill running endurance of WT and mKO mice before (pre) and after (post) 4-weeks of voluntary wheel training (adjusted for training volume) using graded intensity. Two-way ANOVA, interaction effect, Sidak post-hoc effect.

STR signaling genes are expressed in different mouse skeletal muscles (Extended Data Fig.1a), in mouse myofibers *in vivo* (Fig.1a and Extended Data Fig.1b), and in mouse and human primary myocyte cultures (Extended Data Fig.1c,d). The active expression of Tas1r2 in adult skeletal myofibers was further confirmed using reporter mice (Fig.1b and Extended Data Fig.1e) to partially circumvent issues with TAS1R2 antibody reliability (Supplementary Fig.1a).

**Extended Data Figure 2.**
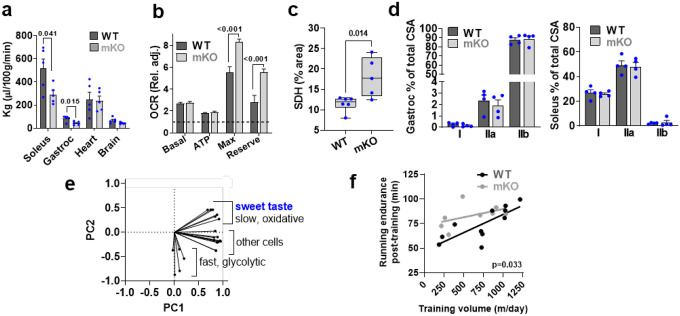
Deletion of TAS1R2 in skeletal muscle induces improvements in skeletal muscle fitness. **(a)** Rate of glucose uptake in skeletal muscles, heart and brain of WT and mKO mice during moderate intensity treadmill running. T-test. **(b)** Relative oxygen consumption rate (OCR) of differentiated myocytes from WT and mKO mice. Dotted line shows leak respiration adjusted to 1. T-test **(c)** Succinate dehydrogenase (SDH) density of WT and mKO gastrocnemius muscles. T-test. **(d)** Relative fiber-type cross-sectional area (CSA) of WT and mKO gastrocnemius (left) and soleus (right) muscles. T-test. **(e)** Principal component analysis (PCA) biplot of skeletal muscle fiber-type gene expression markers and sweet taste receptor signaling genes (Tas1r2, Tas1 r3, and Gnat3). **(f)** Simple linear regression between training volume and post-training treadmill running endurance (adjusted for pre-training) of WT and mKO mice using graded intensity. Elevations contrast p value.

To study the role of TAS1R2 in skeletal muscle, we developed mice with muscle-specific deletion of Tas1r2 (**mKO**) (Supplementary Fig.1b; see methods) followed by a mouse with transgenic overexpression of the human TAS1R2 in skeletal muscle (mTg) (Supplementary Fig.1c,d; see methods). mKO mice had similar body mass (Extended Data Fig.1f) but increased lean mass compared to WT controls, while reconstitution of TAS1R2 expression in mKO mice (i.e., mTg) restored the WT phenotype (Fig.1c). Consistent with the effects on lean mass, skeletal muscle mass (Fig.1d and Extended Data Fig.1g) and myofiber cross sectional area (Fig.1e) was increased in mKO mice and these effects were independent of total body mass (Extended Data Fig.1h), total number of myofibers and nuclei number (Extended Data Fig.1 i,j). Increased muscle mass in mKO mice was accompanied by increased muscle strength (Fig. 1f) and running endurance (Fig.1g) compared to mTg and WT controls. Moreover, mKO mice showed increased running efficiency (i.e., lower O_2_ consumption) during moderate intensity exercise (Fig. 1h) consistent with reduced glucose clearance in skeletal muscle (Extended Data Fig. 2a) and reliance on glucose oxidation (Fig.1i), which suggests improved mitochondrial function. Intact myofibers (Fig.1j) or primary myocyte cultures (Extended Data Fig.2b) from mKO mice had higher maximum coupled respiration compared to WT controls. Moreover, succinate dehydrogenase (SHD)-positive oxidative muscle fibers (Extended Data Fig.2c), respiratory chain complex protein content (Fig.1k), and mitochondrial density (Fig.1l) were also increased in mKO muscles. The enhanced mitochondrial and oxidative profile of mKO muscles was not due to differences in fiber type distribution (Extended Data Fig.2d). However, principal component analysis (PCA) showed that STR signaling gene expression strongly correlated with genes expressed in Type I and IIa oxidative myofibers (Extended Data Fig.2e). Finally, we assessed the performance of mKO mice after a moderate exercise training program. Four weeks of exercise wheel training improved running endurance in both genotypes, but mKO mice had better responses than WT (i.e., training × genotype interaction, p=0.010), independent of baseline performance and training volume (Fig.1m and Extended Data Fig.2f). Taken together our data suggest that the deletion of TAS1R2 sugar sensor in skeletal muscle increases muscle mass and fitness and improves adaptations to exercise training.

### Deletion of Tas1r2 rescues muscle dysfunction associated with obesity and aging

**Extended Data Figure 3.**
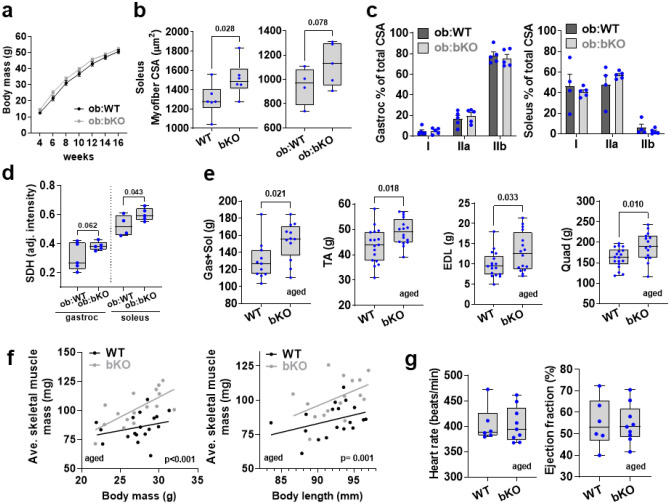
Effects of TAS1R2 deletion in muscle mass and fitness of obese or aged mice. **(a)** Total body mass gain over time in ob:WT and ob:bKO mice. Two-way ANOVA. **(b)** Myofiber cross-sectional area (CSA) of soleus muscle of WT and bKO mice (left) or ob:WT and ob:bKO mice (right). T-test. **(c)** Relative fiber-type cross-sectional area (CSA) of ob:WT and ob:bKO gastrocnemius (left) and soleus (right) muscles. T-test. **(d)** Succinate dehydrogenase (SDH) levels of WT and mKO gastrocnemius and soleus muscles. T-test. **(e)** Mass of gastrocnemius, soleus, tibialis anterior (TA), extensor digitorum longus (EDL), and quadriceps muscles of WT and bKO mice. T-test. **(f)** Simple linear regression between average mass of muscles and total body mass (left) or body length (right) of aged WT and bKO mice. Elevations contrast p value. **(g)** Heart rate (left) and ejection fraction (right) of aged WT and bKO mice during echocardiography. T-test.

**Figure 2.**
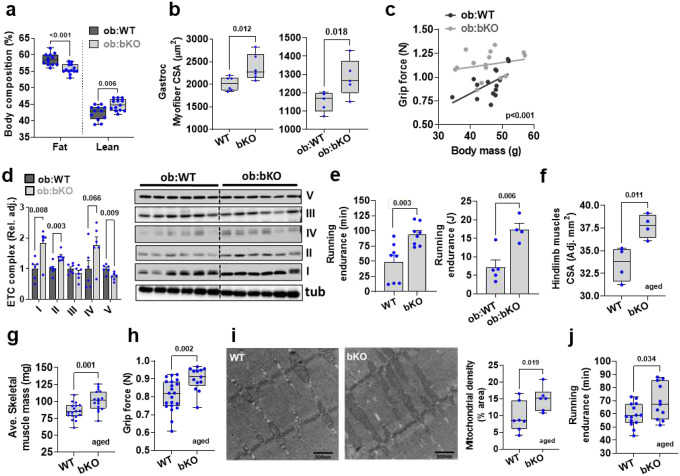
Effects of TAS1R2 deletion in muscle mass and fitness of obese or aged mice. **(a)** Relative fat and lean mass in ob/ob mice (ob:WT) and ob/ob mice with a whole-body deletion of Tas1r2 (ob:bKO). T-test. **(b)** Myofiber cross-sectional area (CSA) of gastrocnemius muscle of WT and bKO mice (left) or ob:WT and ob:bKO mice (right). T-test. **(c)** Simple linear regression between total body mass and forelimb grip force of WT and mKO mice. Elevations contrast p value. **(d)** Immunoblotting of mitochondrial electron transport chain (ETC) proteins of ob:WT and ob:bKO muscles. T-test. **(e)** Treadmill running endurance of WT and bKO mice (left) ob:WT and ob:bKO mice (right) using constant intensity. T-test. **(f)** Cross-sectional area (CSA) of aged WT and bKO hindlimb muscles using computed tomography. T-test. **(g)** Average mass of muscles in aged WT and bKO mice. T-test. **(h)** Forelimb grip force of aged WT and bKO mice. T-test. **(i)** Mitochondrial structure and density of aged WT and mKO muscle using transmission electron microscopy (TEM). T-test. **(j)** Treadmill running endurance of aged WT and bKO mice using constant intensity. T-test.

Next, we investigated the effects of TAS1R2 on preserving muscle function in ob/ob mice, a genetic model of obesity that manifests muscle atrophy^24^. Genetic deletion of TAS1R2 in ob/ob mice (ob:bKO) displayed reduced fat mass and increased lean mass (Fig.2a) despite similar gains in total body mass (Extended Data Fig.3a). Like in mKO mice, myofiber CSA was higher in bKO and ob:bKO mice compared to lean (WT) and obese (ob:WT) controls (Fig.2b Extended Data Fig.3b). However, deletion of TAS1R2 did not affect fiber type distribution in muscles of ob/ob mice (Extended Data Fig.3c). Consistent with the muscle-specific phenotype in mKO mice, ob:bKO mice had increased grip strength independent of body mass (Fig.2c), increased mitochondrial protein content (Fig.2d) and SHD levels (Extended Data Fig.3d), and prolonged running endurance (Fig.2e).

Aging is also strongly associated with muscle loss and dysfunction^25^. Compared to aged (27-month-old) WT controls, aged bKO mice had increased muscle size and mass (Fig.2f,g and Extended Data Fig.3e), independent from total body mass or body size (Extended Data Fig.3f). The effects on muscle mass were accompanied by improvements in muscle strength (Fig. 2h). Aged bKO mice also had increased mitochondrial density (Fig.2i), and improved exercise endurance (Fig. 2j) that cannot be attributed to differences in cardiac function (Extended Data Fig.3g). Taken together the findings suggest that, like in young lean mice, deletion of Tas1r2 in obese or aged mice displays improvements in muscle mass and function.

### Partial loss-of-function of TAS1R2 in humans recapitulates the muscle phenotype of mKO mice in response to exercise training

**Extended Data Figure 4.**
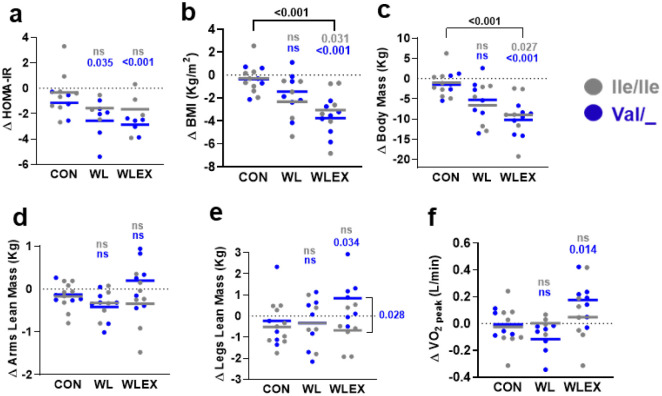
Effects of TAS1R2 loss-of-function variant on responses to exercise training. Effects of TAS1R2-Ile191Val variant in older obese individuals subjected to 6-months of diet-induced weight loss with exercise training (WLEX), diet-induced weight loss alone (WL), or education control (CON). Ile/Ile (TAS1R2 normal function) or Val/_ (TAS1R2 partial loss-of-function). Changes (Δ) before and after the interventions are shown. **(a)** Homeostatic model assessment for insulin resistance (HOMA-IR). **(b)** Body mass index (BMI). **(c)** Body mass. **(d, e)** Arms and legs lean mass. **(f)** Peak oxygen consumption (VO2). Statistics: (a-f) Values are adjusted for sex, age and diabetic status with a two-way ANCOVA. Brackets indicate post-hoc effects between treatment groups or between genotypes within a group. Non-significant effects (p<0.05) are not shown. Gray font (Ile) or blue font (Val) p-values show contrasts between the corresponding genotype within a treatment group and the control (CON) group. ns, non-significant. P-values with brackets indicate differences between genotypes. #, p<0.05 between genotype and control (CON) group. ns, non-significant.

**Figure 3.**
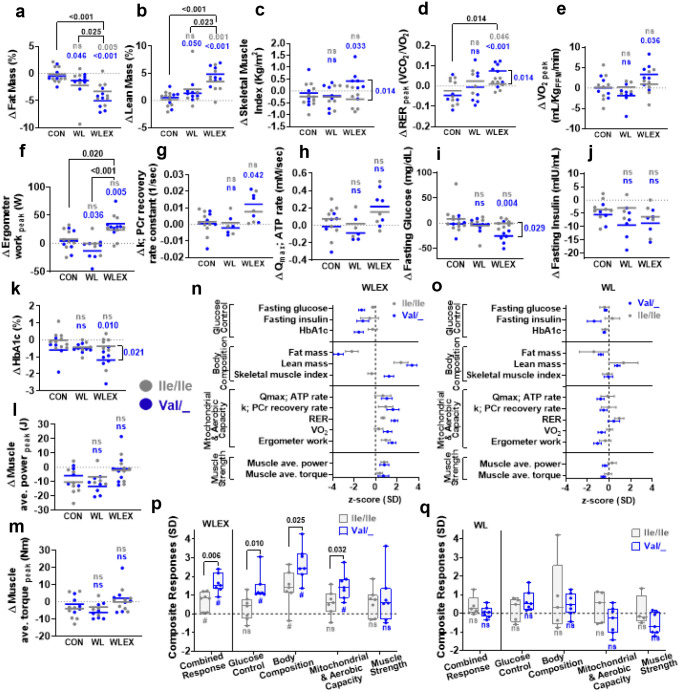
Effects of TAS1R2 loss-of-function variant on responses to exercise training. Effects of TAS1R2-Ile191Val variant in older obese individuals subjected to 6-months of diet-induced weight loss with exercise training (WLEX), diet-induced weight loss alone (WL), or education control (CON). Ile/Ile (TAS1R2 normal function) or Val/_ (TAS1R2 partial loss-of-function). Changes (D) before and after the interventions are shown. **(a)** Fat mass. **(b)** Lean mass. **(c)** Skeletal muscle index. **(d)** Peak respiratory exchange ratio (RER), **(e)** peak oxygen consumption (VO2), and **(f)** peak work during cycle ergometer testing. **(g)** Phosphocreatine (PCr) recovery rate constant (k), and **(h)** maximum oxidative ATP synthesis rate (Qmax) in muscles *in vivo* using phosphorus magnetic resonance spectroscopy (31P-MRS). **(i, j)** Fasting blood glucose and insulin. **(k)** Glycated hemoglobin (HbA1c). **(l, m)** Muscle average power and torque. **(n, o)** Control group-adjusted z-scores of changes (D) for the Ile and Val participants within the WLEX and WL treatment groups. **(p, q)** Composite responses of z-scores for all variables within the four physiological categories. Combined responses represent average of all four physiological composites. Statistics: (a-m) Values are adjusted for sex, age and diabetic status with a two-way ANCOVA. Brackets indicate post-hoc effects between treatment groups or between genotypes within a group. Non-significant effects (p<0.05) are not shown. Gray font (Ile) or blue font (Val) p-values show contrasts between the corresponding genotype within a treatment group and the control (CON) group. ns, non-significant. (p, q) Welch’s adjusted T. p-values with brackets indicate differences between genotypes. #, p<0.05 between genotype and control (CON) group. ns, non-significant.

Ablation of TAS1R2 in mice produced a robust exercise-trained phenotype and preserved muscle function in obese or aged mice. To translate this finding to humans, we evaluated the impact of *TAS1R2-Ile191Val* partial loss-of function variant^26,27^ in older obese individuals who participated in a 6-month trial of diet-induced weight loss with exercise training (WLEX), diet-induced weight loss alone (WL), or an education control (CON)^28,29^. Participants within each group were retrospectively genotyped and classified as Ile/Ile (i.e., TAS1R2 conventional function) or Val/_(i.e., TAS1R2 partial loss-of-function). We examined parameters that are directly comparable to those assessed in mice: glucose control, body composition, mitochondrial and aerobic capacity, and muscle strength before and after the interventions (Supplementary Fig.2a,b). The WL intervention caused a drop in body mass in the combined cohort of Ile/Ile and Val/_ carriers (Extended Data Table.1). Addition of exercise training (WLEX) caused further, but modest, improvements in body composition (Fig.3a,b and Extended Data Fig.4b,c) and aerobic performance (Fig.3d,f).

At baseline (i.e., pre-intervention), there were no major genotype differences in the assessed variables within or between intervention groups (Extended Data Table.2) and the TAS1R2-Ile191Val allele frequency and distribution was typical (Extended Data Table.3). In the WLEX treatment group, the Val/_ carriers had enhanced and extensive responses to exercise training compared to Ile/Ile (Fig.3c,d,i,k, Extended Data Table.4 and Extended Data Fig.4e). In addition, partial loss-of-function of TAS1R2 also unveiled significant effects on several other outcomes that were otherwise masked in the WLEX group. For instance, Val/_ carriers exhibited increased skeletal muscle mass (Fig.3c and Extended Data Table.4 and Extended Data Fig.4e), improved mitochondrial capacity (Fig.3g) and aerobic performance (Fig.3e,f and Extended Data Fig.4f), and demonstrated significant reductions in fasting glucose, HbA1c^27^ (Fig.3i,k), and HOMA-IR (Extended Data Table.4 and Extended Data Fig.4a) compared to CON group. These effects were absent in the Ile/Ile counterparts.

The magnitude of responses varies between participants and individual variables. Therefore, we considered cumulative effects of all measured outcomes to rank the performance of participants from both genotypes for each of the four physiological categories (Fig.3n,o). In the WLEX group, Val/_ carriers scored significantly higher in all categories compared to their Ile/Ile counterparts (Fig.3p). Muscle strength was the only category that did not improve for both genotypes (Fig.3p), but overall, the WLEX group performed significantly better than the WL group (muscle strength WLEX vs. WL, p=0.006; Fig.3p,q). This indicates that diet-induced weight loss may have slightly compromised muscle performance, but concomitant exercise training partially offset this effect. Most notably, when the combined performance from all four categories was considered, most Val/_ participant in the WLEX group responded substantially better to the exercise intervention than any of the Ile/Ile participant, revealing a large effect size difference (Cohen’s d=−1.79; Fig.3p and Source Data F3). Our results demonstrate prominent genotype-dependent adaptations to exercise training which, like in mice, show that TAS1R2 partial loss-of-function in humans confers beneficial effects associated with muscle mass and function.

### TAS1R2 regulates NAD levels in skeletal muscle

**Extended Data Figure 5.**
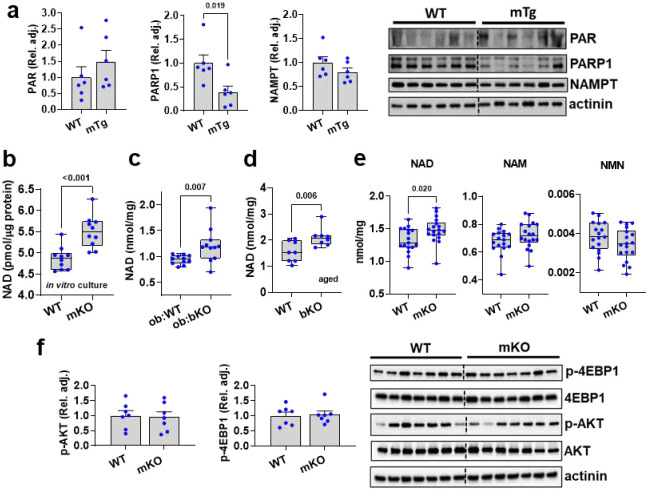
TAS1R2 regulates skeletal muscle NAD levels. **(a)** Immunoblotting of poly(ADP)-ribosylation (PAR), PARP1, and NAMPT in WT and mTg muscles. T-test. **(b)** NAD concentration in primary myocyte cultures from WT and mKO muscles. T-test. **(c)** NAD concentration in ob:WT and ob:bKO muscles. T-test. **(d)** NAD concentration in aged WT and bKO muscles. T-test. **(e)** Quantitative targeted nucleotide analysis (NAD, NAM, and NMN) in WT and mKO muscles using LC/MS. T-test. **(f)** Immunoblotting of p-EBP1, 4EBP1, p-AKT, and AKT in WT and mKO muscles. T-test.

**Figure 4.**
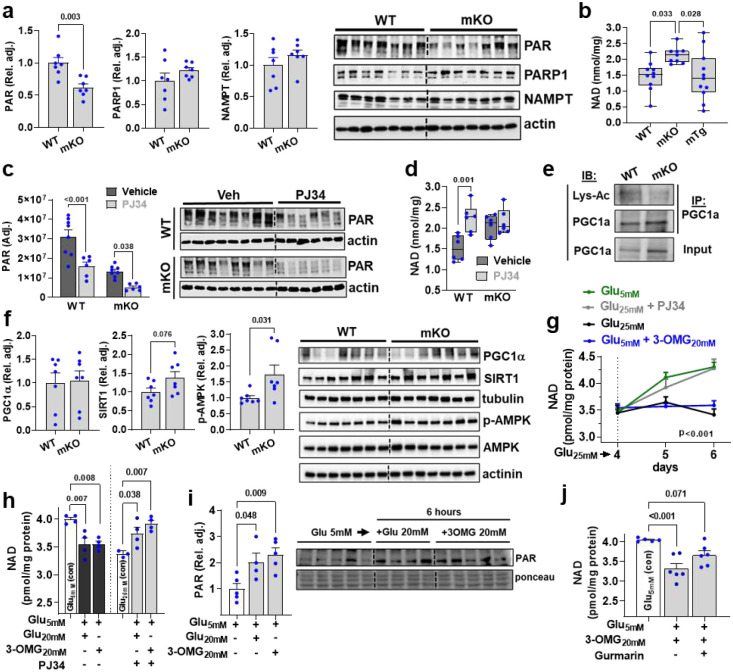
TAS1R2 regulates skeletal muscle NAD levels. **(a)** Immunoblotting of poly(ADP)-ribosylation (PAR), PARP1, and NAMPT in WT and mKO muscles. T-test. **(b)** NAD concentration in WT, mKO and mTg muscles. One-way ANOVA, Sidak post-hoc effect. **(c)** Immunoblotting of PAR in WT and mKO muscles in response to 5-days of treatment with the PARP1/2 inhibitor, PJ34. Two-way ANOVA, Sidak post-hoc effect. **(d)** NAD concentration in WT and mKO muscles in response to 5-days of treatment with the PARP1/2 inhibitor, PJ34. Two-way ANOVA, Sidak post-hoc effect. **(e)** Immunoblotting (IB) of acetylated PGC1a (Lys-AC) in WT and mKO muscle lysates immunoprecipitated (IP) with total PGC1a. **(f)** Immunoblotting of PGC1a, SIRT1, p-AMK, and AMPK in WT and mKO muscles. T-test. **(g)** NAD concentration in C2C12 cells that were maintained in high glucose (Glu 25mM) for 4 days and then subjected to 2 additional days in high glucose (Glu 25mM) with or without PJ34, or low glucose (Glu 5mM) with or without 3-OMG (3-OMG 20mM). Two-way ANOVA, interaction effect (n=12–15). **(h)** NAD concentration in C2C12 cells that were maintained in low glucose (Glu 5mM) and were subsequently spiked with additional glucose (Glu 20mM) or 3-OMG (3-OMG 20mM) with or without PJ34 (PARP1/2 inhibitor) for 6 hours. C2C12 cells were maintained to either Glu 5mM or Glu 25mM as reference controls (con; white bars). One-way ANOVA, Sidak post-hoc effect. **(i)** Immunoblotting of PAR in C2C12 cells that were maintained in Glu 5mM and then were subsequently spiked with additional Glu 20mM or 3-OMG 20mM for 6 hours. One-way ANOVA, Sidak post-hoc effect. **(j)** NAD concentration in C2C12 cells that were maintained in Glu 5mM and then were subsequently spiked with additional 3-OMG 20mM with or without gurmarin (Tas1r2 inhibitor). One-way ANOVA, Sidak post-hoc effect.

Mice with genetic ablation of PARP^13,14^ or TAS1R2^23^ exhibit striking phenotypic similarities (Fig. 1,2), which suggested that the functions of these 2 proteins might be intertwined. mKO muscles had reduced protein poly(ADP)-ribosylation (PAR), a measure of PARP activity (Fig.4a), while PAR levels in WT and mTG muscles were comparable (Extended Data Fig.5a). mKO muscles also had increased NAD levels compared to WT controls and mTg muscles (Fig.4b), consistent with reduced PARP activity^13,14,30^. The TAS1R2 genotype effect on NAD was recapitulated in murine primary myocyte cultures and in muscles from ob:bKO and aged bKO mice (Extended Data Fig.5b,c,d). LC/MS analysis also showed elevated NAD levels in mKO muscles whereas the concentrations of NAD precursors^30^, NAM and NMN (Extended Data Fig.5e), were unchanged. Accordingly, the protein levels of the rate liming enzyme of NAD synthesis, nicotinamide phosphoribosyl transferase (NAMPT), were also comparable in WT and mKO muscle (Fig.4a). Pharmacological inhibition of PARP1/2 with PJ34 increased muscle NAD in WT mice^14^ at levels similar to vehicle-treated mKO, but it failed to further increase NAD in mKO muscles, which suggests that the lower PAR levels in mKO muscles were sufficient to maximize NAD (Fig.4c,d). mKO muscles had reduced peroxisome proliferator activated receptor-γ coactivator-1α (PGC1α) acetylation (Fig.4e) which is consistent with the increased NAD and a modestly elevated SIRT1 protein levels^13,14^ (Fig.4f). The activation of NAD-SIRT1 axis was also accompanied by a moderate activation of AMPK^10,31^ (Fig.4f), but other related pathways remained unchanged (Extended Data Fig.5f). Taken together, our data indicate that TAS1R2-mediated regulation of NAD is coupled, at least in part, to PARP1 activity.

### STR link ambient glucose sensing to the regulation of NAD in skeletal muscle

C2C12 muscle cells that were either shifted to low glucose^10,32^ (Fig.4g, green trace) or treated with PJ34^13^ (Fig.4g, gray trace) for 2 days increased NAD levels compared to cells maintained in high glucose alone (Fig.4g, black trace). Notably, when 3-O-methylglucose (3-OMG) - a nonmetabolizable glucose analogue^33^ that has equal affinity for TAS1R2 as glucose^34^ - was added to cells maintained in low glucose, NAD levels were reversed to those seen under the high glucose condition (Fig.4g, blue trace). As with the chronic treatment above, C2C12 cells in low glucose spiked with equal molarity glucose or 3-OMG for 6h, displayed a comparable drop in NAD levels (Fig.4h, black bars) that was associated with a corresponding increase in PARP activity (Fig.4i). Consequently, inhibition of PARP with PJ34 reversed NAD levels in both treatments (Fig.4i, gray bars). Notably, the drop in NAD levels in response to 3-OMG addition was partially prevented with gurmarin, a STR inhibitor (Fig.4j). Taken together, these findings support a putative link between glucose-induced activation of STR-PARP axis and the regulation of NAD in muscle cells.

### STR activate ERK2-PARP1 axis in skeletal muscle

**Extended Data Figure 6.**
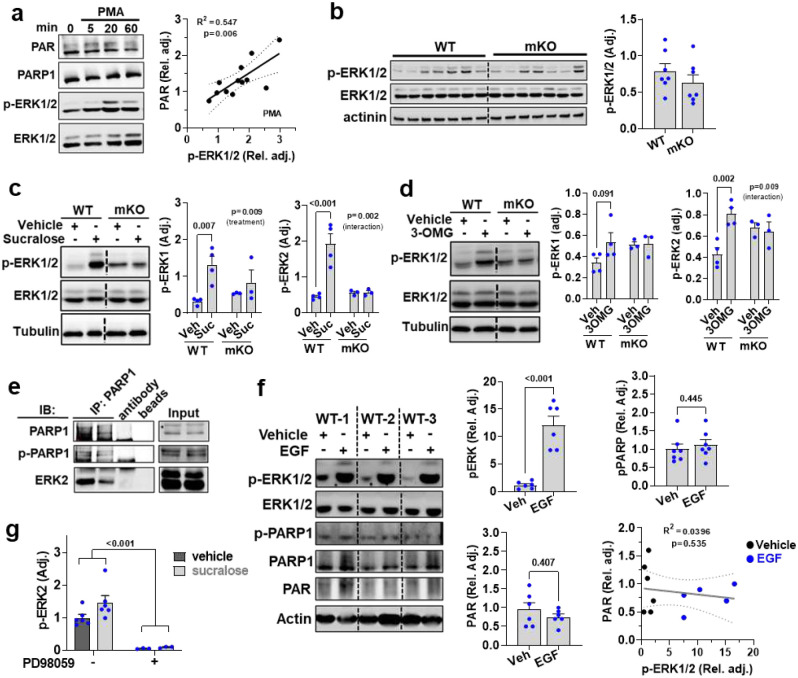
TAS1R2 activates the ERK2-PARP1 axis in skeletal muscle. **(a)** Immunoblotting of PAR, PARP1, p-ERK1/2 in C2C12 cells following treatment with the ERK1/2 activator, PMA (left). Simple linear regression of p-ERK1/2 with PAR in PMA-treated C2C12 cells (right); Slope p-value. **(b)** Immunoblotting of p-ERK1/2 in WT and mKO muscles. T-test. **(c)** Immunoblotting of p-ERK1/2 in response to intramuscular injection of sucralose or vehicle in WT and mKO muscles. Two-way ANOVA interaction effect and Sidak post-hoc effect. **(d)** Immunoblotting of p-ERK1/2 in response to intramuscular injection of 3-OMG or vehicle in WT and mKO muscles. Two-way ANOVA interaction effect and Sidak post-hoc effect. **(e)** Immunoblotting (IB) of p-PARP and ERK2 in WT nuclear muscle lysates immunoprecipitated (IP) with PARP1. (**f**) Immunoblotting of p-ERK, p-PARP1, and PAR in response to intramuscular injection of EGF or vehicle in WT muscles; T-test. Simple linear regression of p-ERK1/2 with PAR in Vehicle- or EGF-treated muscles; Slope p-value. **(g)** Immunoblotting of p-ERK2 in C2C12 cells following treatment with sucralose with or without PD98059; Two-way ANOVA, inhibitor treatment effect (brackets).

**Figure 5.**
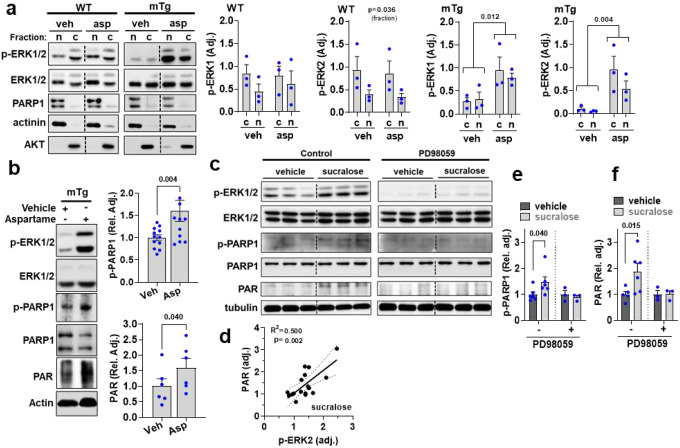
TAS1R2 activates the ERK2-PARP1 axis in skeletal muscle. **(a)** Immunoblotting of p-ERK1/2 in response to intramuscular injection of aspartame (asp) or vehicle (veh) in nuclear (n) or cytoplasmic (c) fractions of WT and mTg muscles. Two-way ANOVA fraction effect and treatment effect (brackets). **(b)** Immunoblotting of p-PARP1 and PAR in response to intramuscular injection of aspartame or vehicle of WT and mTg muscles. T-test. **(c)** Immunoblotting of p-ERK, p-PARP1, and PAR in C2C12 cells treated with sucralose or vehicle, with or without the ERK1/2 inhibitor, PD98059. **(d)** Simple linear regression of p-ERK2 with PAR in sucralose-treated C2C12 cells; Slope p-value. **(e)** Immunoblotting of p-PARP1 in C2C12 cells treated with sucralose or vehicle, with or without the ERK1/2 inhibitor, PD98059. T-test. **(f)** Immunoblotting of PAR in C2C12 cells treated with sucralose or vehicle, with or without the ERK1/2 inhibitor, PD98059. T-test.

STR trigger phospholipase C (PLC)-mediated signaling cascades^18–20,22^, including the extracellular signal-regulated protein kinases (ERK) axis^35^. ERK2 can activate PARP1 in neurons^36–38^, so we activated ERK1/2 in muscle cells with PMA and found it also produced proportional increases in PARP activity (Extended Data Fig.6a). Baseline ERK1/2 phosphorylation was similar between genotypes (Extended Data Fig.6b), so to test the STR-ERK-PARP1 axis in skeletal muscle we activated STR via intra-muscular injection of sucralose or 3-OMG (Extended Data Fig.6c,d) which rapidly induced ERK1/2 phosphorylation in WT, but not mKO muscles. Similarly, aspartame, a human-specific TAS1R2 agonist (Supplementary Fig.1e), activated ERK1/2 in “humanized” mTg muscles, but not in WT muscles (Fig.5a). Since PARP1 is nuclear, we determined the activation status and localization of ERK1/2 in our models. ERK1, but predominately ERK2, was induced in both the cytoplasm and nucleus in response to aspartame in mTg muscles (Fig.5a). ERK2 was also co-immunoprecipitated with PARP1 in muscle nuclear isolates, revealing these two proteins interact (Extended Data Fig.6e). Using an ERK2-specific phospho-PARP1 antibody (Extended Data Fig.6e), we found increased ERK2-mediated PARP1 phosphorylation^36^ and PARP activity in mTg muscles treated with aspartame (Fig.5b). In contrast, treatment with EGF, which also robustly activates the ERK pathway^39^, did not increase PARP1 phosphorylation or its activity in skeletal muscle (Extended Data Fig.6f). In addition, sucralose induced pERK2 in C2C12 cells (Fig.5c) and caused proportional increases in PARP activity (Fig. 5d) thus mirroring the effects of PMA (Extended Data Fig.6a). Direct inhibition of ERK1/2 with 2-(2-amino-3-methoxyphenyl)-4H-1-benzopyran-4-one (PD98059; PD) (Extended Data Fig.6f) blocked sucralose-induced PARP1 phosphorylation and PAR (Fig.5e,f), further supporting the existence of a TAS1R2-ERK2-PARP1 signaling axis. Taken together, these data indicate that STR signaling in skeletal muscle targets PARP1, at least in part, through ERK1/2 activation.

## DISCUSSION

All cells possess glucose sensing mechanisms that link energy availability to cellular processes governing homeostasis. These mechanisms rely on cell uptake of glucose and its transformation to metabolic products that are detected by cellular sensors^40^. In this study, we showed that TAS1R2, the sugar-sensing GPCR originally shown to mediate sweet taste perception on the tongue^41^, is a plasma membrane glucose sensor in skeletal muscle that regulates muscle mass and fitness in mice and humans.

TAS1R2 signaling in skeletal muscle activated PARP1 which consumed NAD and produced ADP-ribose polymers. On the other hand, attenuating the TAS1R2-PARP1 axis increased the NAD level in skeletal muscles. PARP1 inhibition has been shown to raise NAD levels and cause SIRT1 activation and de-acetylation of PGC1α^13,14^. TAS1R2 loss-of-function phenocopied the PARP1 loss-of-function in mice which included improved muscle fitness (i.e., mitochondrial capacity, muscle strength and running endurance) arising from activation of the NAD-SIRT1 axis and AMPK^10,31^. The importance of this novel signaling cascade involving TAS1R2 in skeletal muscle is supported by the ability of the TAS1R2 loss-of-function to mitigate the functional decline of muscles associated with obesity or aging. Mechanistically, we showed that stimulation of STR in skeletal muscle induced ERK2, which then activated PARP1 through specific phosphorylation^36–38^. The ERK2-PARP1 interaction, which was previously unknown in skeletal muscle, is not indiscriminate. It is likely coupled to specific extracellular stimuli, including the activation of TAS1R2 receptor. Our findings also revealed that NAD levels *in vitro* could be regulated by glucose dependent on the activation of STR-PARP1 axis. Therefore, we demonstrate for the first time that the well-known metabolic adaptions of NAD in response to ambient glucose fluctuations^9,10^ do not solely rely on glucose uptake and metabolism but can also involve nutrient sensing GPCRs. In fact, the affinity values of glucose for TAS1R2^42^ coincide with the physiological blood glucose concentrations, suggesting possible physiological ramifications in response to feeding states.

One of the main phenotypic attributes of the mKO mice, increased skeletal muscle mass, was not displayed by the PARP1 loss-of-function mice. This finding suggested that TAS1R2 signaling intersected with additional pathways that regulate anabolic processes. STR activation during states of energy surplus (e.g., postprandial) might serve as a bioenergetic checkpoint to curtail redundant muscle growth and/or futile energy cycles. Despite nutrient abundance, STR-deficient muscles do not constrain the PARP1-NAD-SIRT1 axis and anabolism, thereby enabling further positive adaptations in muscle fitness and mass, respectively. In summary, TAS1R2-dependent pathways inform myocytes about the peripheral energy status to modulate, along with other known mechanisms, the intracellular processes that govern muscle structure and function.

Genetic manipulations in mice are a powerful approach to decipher the function of genes and their role in the development of disease. However, the resulting mouse phenotypes often do not translate with fidelity to human pathophysiology due to fundamental mechanistic differences and populational heterogeneity inherent to human biology. However, the bKO mice and the partial loss-of-function TAS1R2-Ile191Val human variant (rs35874116) both displayed reduced glucose excursions during an OGTT^20,26^. Hence, we used the TAS1R2-Ile191Val variant to translate the mouse TAS1R2 findings to humans. We performed a retrospective analysis of a randomized interventional study conducted in older obese individuals as this study specifically assessed several measured variables associated with muscle mass, strength, mitochondrial capacity and aerobic performance^28,29^. This approach was necessary to enable direct comparisons between the human and rodent loss-of-function phenotypes.

The exercise intervention (WLEX) produced only modest to no improvements in the associated outcomes in the whole study population, a fact attributed to the heterogeneity of responses to exercise training among participants^29,43^. However, retrospective consideration of the Ile191Val variant as a factor revealed characteristics that phenocopied the mKO mice. Specifically, participants with TAS1R2 partial loss-of-function (Val/_) exhibited increased muscle mass and improved mitochondrial capacity and aerobic performance in response to the exercise intervention, unlike participants with “wild type” TAS1R2 function (Ile/Ile). The exercise training protocol was primarily focused on improving aerobic performance, which likely explains why muscle strength remained unchanged for both genotypes. Notably, the genotype differences in response to the exercise intervention were comprehensive. An analysis of cumulative effects revealed partitioning between genotypes, whereas Ile/Ile participants were low or no responders to exercise, and Val/_ participants were high responders. The genetic and physiological factors contributing to the heterogeneous response to exercise are under ongoing investigation^44^. Therefore, the predictive value of this SNP should be further tested though specialized interventional studies. Finally, Val/_ carriers also showed significant improvements in glucose control. Specifically, the lower HbA1c in Val/_ carriers is consistent with previous observations^27^. These outcomes were not seen in the Ile/Ile counterparts, even though both genotype groups had similar baseline glycemic profiles and lost the same amount of body mass in response to the intervention. It is not apparent which tissues expressing STR contributed to the effects on glucose control, but inhibition of TAS1R2 signaling might have independent effects on glucose homeostasis unrelated to skeletal muscle function.

Taken together, our pre-clinical and clinical data suggests that TAS1R2 signaling regulates muscle structure and function. GPCRs are proven targets for pharmacological interventions^45^. Hence, inhibition of TAS1R2 signaling could be a promising therapeutic approach for treating muscle dysfunction associated with aging, obesity or other conditions.

## METHODS

### Animal studies

#### Animals

All animal procedures were performed under the approval of The Ohio State University institutional animal care and use committee (IACUC). Mice were housed on a 12 h light/dark cycle with free access to water and standard diet. C57BL6/6J, routinely obtained every 5^th^ generation from the Jackson lab (Jax# 000664), were used to backcross all mouse models. Tas1r2 Cre knock in (Tas1r2^iCre^) was previously generated as described^1^. tdTomato mice (Td^fl/fl^) were obtained from the Jackson lab (Jax#007914) and crossed with Tas1r2^iCre^ to generate a Tas1r2 reporter (Td^Tas1r2^). Myogenin-Cre mice (Myo^Cre^) were a gift by Dr. Olson. RiboTag mice (Rpl22^fl/fl-HA^) were obtained from the Jackson Lab (Jax# 029977) and crossed with Myo^Cre^ mice to generate a myocyte RiboTag reporter (Ribo^Myo^). Tas1r2 floxed mice (Tas1r2^fl/fl^) were obtained from the KOMP Repository (#CSD25803) and crossed with MyoCre mice to generate muscle-specific knockouts (mKO). Human TAS1R2 transgenic mice (TG) were created by Ingenious targeting lab (NY) by placing a cDNA cassette containing a synthetic CAG promoter, a floxed NEO stop cassette, the consensus human TAS1R2 cDNA, an IRES-EGFP and a BGH polyA on the mouse ROSA26 locus. After backcrossing, the TG allele was extracted into the Tas1r2^fl/fl^ background and further crossed with the mKO line to generate muscle-specific transgenic mice (mTG). Specifically, we routinely bred Cre+ mTG lines with Cre^−^ Tas1r2^fl/fl^ dams to obtain mixed litters of wild type (WT), mKO and mTG experimental mice. Whole body Tas1r2 knockouts (bKO) were a gift from Dr. Zuker. Leptin deficient Lepob mice (ob) were obtained from the Jackson Lab (Jax #000632) and crossed with bKO mice to generate Tas1r2-deficient ob mice (ob:bKO). Mice were used for experimental purposes only after reaching sexual maturity at 12 weeks of age.

#### Mouse genotyping

Mice were ear-punched at weaning and punches were digested with 100μL of 50mM NaOH for 1h at 95°C. Samples were then cooled at room temperature, spun, and neutralized with 20μL of 1M Tris pH8 and 100μL of water. Clear homogenates were subsequently genotyped by PCR and electrophoresis using specific primers and conditions as stated in resource data.

#### Sweetener consumption

An automated phenotyping homecage system (TSE PhenoMaster) was used to perform behavioral and metabolic tests in single-caged mice. The specificity of the TG construct was tested in bTG and WT mice by recording daily liquid intake of water or 5mM aspartame and 600ppm sucralose in 3 subsequent, alternate days.

#### Body composition

Body composition was measured in duplicate using an EchoMRI instrument, calibrated daily with 40.5g of canola oil, and percent composition was calculated after subtraction of free water. Body length and tibia length were measured at harvest using pre-calibrated Traceable digital calipers (Fisher Scientific).

#### Grip tests

Forelimb strength was measured with a 1027SM-D66 grip strength meter (Columbus Instruments). Mice forelimbs were positioned evenly on a slanted grid and mice were pulled horizontally from the tail for 5 times, spaced at least 30 seconds to avoid exhaustion. Test were performed in duplicate in different days and values were averaged.

#### Running endurance

Running endurance was performed between 12 and 2 p.m. in 5h fasted male mice using an Exer 6 treadmill (Columbus Instruments). All mice were accustomed to the treadmill for at least 2 sessions of acclimatization. The standard acclimatization in lean mice consisted in sessions of 5min-0°−0m/min, 10min-10°−0m/min and 15min-10°−5m/min, while OB mice were acclimatized with short sessions of 5min-0°−0m/min (without incline). Endurance was tested with four different protocols to accommodate each mouse model. For young WT and mKO mice, endurance was tested with a constant speed protocol, consisting of 5min-0°−10m/min, 5min-10°−10m/min, 5min-10°−15m/min, and 10°−20m/min until exhaustion. In ob:WT and ob:bKO mice, endurance was measured without incline in a single step of 0°−10m/min until exhaustion. In aged mice (WT and bKO, endurance was tested with a mild ramp protocol to accommodate mice with gait problems, starting 5min-0°−5m/min, followed by 5min-10°−5m/min, 5min-10°−10m/min and then increasing 1m/min every 5min until exhaustion. Running endurance for WT and mKO mice submitted to an exercise program (see Exercise Training below) was evaluated with a more aggressive ramp protocol, starting 5min-0°−10m/min, followed by 5min-0°−20m/min, 5min-10°−20m/min and then increasing 1m/min every 5min until exhaustion.

#### Exercise training

Mice were housed individually for 30 days in standard cages containing an in-cage running wheel (STARR Life Sciences) to provide voluntary access to physical exercise. Wheel revolutions through glass reed switches and recorded every 30min with VitalView data acquisition software (STARR Life Sciences). Revolutions were transformed to distance in cm through an estimated internal diameter of 11.0998 cm. The distance run per day was averaged to estimate the training volume.

#### Exercise calorimetry

Gas exchange during treadmill exercise was performed as previously described^2^. Briefly, mice were acclimated to an enclosed, motorized treadmill connected to O_2_ and CO_2_ sensors (Columbus Instruments, Columbus, OH) by performing an acclimation run (10 m/min for 10 min). Two days later, mice were placed back in the enclosed treadmill and were allowed to acclimate for 30 min. The exercise test then began with a 2 min run at 10 m/min at a 0% incline which was then adjusted to 20 m/min at a 10% incline for 60 min. Mice were encouraged to run using an electric shock grid at the back end of the treadmill (1.5 mA, 200 msec pulses, 4 Hz). Oxygen consumption (VO_2_) and carbon dioxide production (VCO_2_) in ml/(kg·min) were continuously measured with an airflow rate of 1 m/min. RER was calculated as VCO_2_/VO_2_.

#### Peripheral glucose uptake during exercise

Tissue glucose clearance was determined as previously described^2^. Indwelling catheters were surgically implanted in the carotid artery and jugular vein for blood sampling and infusions, respectively. Following a 5-day recovery period, mice were acclimated to an open, motorized treadmill (Columbus Instruments, Columbus, OH) by performing a 10 min run at 10 m/min. Two days later, 5h-fasted mice were placed in the treadmill and underwent a 30 min exercise bout at 16 m/min, 0% incline. Mice were encouraged to run using an electric shock grid at the back end of the treadmill (1.5 mA, 200 msec pulses, 4 Hz). At t=5 min into the run a 12 mCi bolus of 2[^14^C]-deoxyglucose (2[^14^C]DG) was administered via the jugular vein catheter. Blood samples were collected via the arterial catheter at t=7, 10, 15, 20, and 30 min to measure blood glucose (AccuChek, Roche) and plasma 2[^14^C]DG. At t=30 min, mice were anesthetized with an arterial infusion of sodium pentobarbital, and tissues were rapidly excised, snap frozen, and stored at −80°C until processed. Plasma 2[^14^C]-DG and tissue 2[^14^C]-DG-6-phosphate (2[^14^C]DGP) were measured as described previously^3,4^. After deproteinization with barium hydroxide [Ba(OH)2, 0.3 N] and zinc sulfate [ZnSO4, 0.3 N], plasma 2[^14^C]DG radioactivity was determined by liquid scintillation counting (Packard TRI-CARB 2900TR) with Ultima Gold (Packard) as scintillant. Muscle samples were weighed and homogenized in 0.5% perchloric acid. Homogenates were centrifuged and neutralized with KOH. One aliquot was counted directly to determine 2[^14^C]DG and 2[^14^C]DGP radioactivity. A second aliquot was treated with Ba(OH)_2_ and ZnSO_4_ to remove 2[^14^C]DGP and any tracer incorporated into glycogen and then counted to determine 2[^14^C]DG radioactivity. 2[^14^C]DGP is the difference between the two aliquots. In all experiments, the accumulation of 2[^14^C]DGP was normalized to tissue weight. Tissue glucose clearance (Kg) was calculated as the ratio of tissue 2[^14^C]DGP and the area under the curve of 2[^14^C]DG disappearance from the plasma.

#### Mouse *in vivo* treatments

Administration of 10mg/kg PJ34 was performed IP twice a day for 7 days. For intramuscular sweetener injections, 5h fasted mice were anesthetized with pentobarbital and 10μL of sucralose (100mM), 3OMG (30mM) or aspartame (5mM) were injected with a 20μL Hamilton syringe in the vastus medial using the contralateral leg as saline-injected control. Muscles were dissected exactly 10min after each injection and snap-frozen in liquid nitrogen.

#### Transthoracic echocardiography.

Cardiac function and heart dimensions were determined by 2-dimensional echocardiography using a Visual Sonics Vevo 3100 Ultrasound (Visual Sonics) on mice under anesthesia (initially 2.5% isoflurane mixed with 1 liter per minute 95%O2/5%CO2 and maintained with 1–1.5% isoflurane). A single observer blinded to mouse genotypes performed echocardiography and data analysis. M-mode tracings were used to measure left ventricular dimensions at end diastole and end systole. Ejection fraction (EF%) was calculated by: EF(%) = (EDV − ESV)/EDV, where ESV represents end systolic volume and EDV represents end diastolic volume.

#### CT scanning

In vivo muscle CSA was assessed under 2% isoflurane anesthesia using a SkyScan 1276 computed tomography scan with a 40.9μm resolution. Images were subsequently converted to NIFTI format using FIJI (ImageJ 1.53t) and skin, bones and muscles were reconstructed in ITK SNAP 3.8.0 with before calculating the posterior muscle CSA at 1/3 of the tibia intercondylar notch.

#### Harvest and tissue processing

All tissue harvests were performed in vivo under 165 mg/kg pentobarbital anesthesia using heat pads to sustain body temperature. Muscles were harvested tendon to tendon starting with the left leg and immediately snap-frozen in liquid nitrogen. Samples for RNA extraction were stored at −80 °C until use. Samples for protein determination and metabolomics were lyophilized overnight at −50 °C and 0.5mbar and then pulverized in 2ml Sarstedt tubes containing 2 RNase-free plastic beads in a Precellys homogenizer at 0°C × 7200RPM × 20sec × 15sec pause × 3 cycles.

#### Primary myocyte isolation and culture

Mouse hindlimb muscles were dissected in PBS, sliced into 1mm pieces in HBSS and digested for 45min at 37°C using 400U/ml collagenase and 0.08 U/ml dispase II. Tissues were then triturated by aspiration with a 1ml pipette, incubated for 45min more and the reaction was stopped by addition of pre-warmed pre-plating medium (PPM, 90% low glucose DM EM, 10% FBS, 1x glutamax, 1% penn-strep). Cells were strained, spun at 300g, re-suspended in PPM and incubated into a 100mm tissue dish for 3h. Myoblasts were then released from the culture dish by gentle agitation leaving the adherent fibroblasts behind and the medium was spun at 300g. Pelleted cells were re-suspended in growth medium (GM, 80% Ham’s F-10, 20% FBS, 1x Glutamax, 2.5ng/ml bFGF, 10ng/μL EGF, 1ug/ml insulin, 0.39mg/ml dexamethasone, 1% penn-strep), incubated on collagen-coated T25 flasks, and expanded for 5–6 days until clusters of 2–5 cells appeared. Cells were then purified for a second time (trypsinized in 1:1 PBS, pre-plated with PPM, separated from fibroblasts by agitation, spun at 300g, resuspended in GM) and expanded from T25 to T75 flasks. Cells were differentiated at passage 6 by seeding them 50000 cells into collagen-coated 24-well plates and switching to differentiation media (DM, 98% high glucose DMEM, 2% horse serum, 1x Glutamax, 1% pen-strep) 24h after. NAD was determined after 6 days of differentiation. Primary human skeletal myoblasts were provided by Translational Research Institute Advent Health (Florida, USA). Myoblasts were grown to a confluence of approximately 80% to 90% and differentiated into myotubes as previously described^5^.

#### C2C12 culture and studies

C2C12 (ATCC CRL-1772) were cultured in high glucose DMEM with 10% fetal bovine serum and 1% Penn/Strep using 75cm^2^ flasks. Cells were passaged in 24-well plates for NAD or 6-well plates for signaling studies at a density of 52632 cells/cm^2^ and media was changed to high glucose DMEM with 2% horse serum and 1% Penn/Strep upon confluence to start the differentiation process. NAD experiments were performed by switching to low glucose DMEM 2% horse serum and 1% Penn/Strep at day 4 of differentiation and supplementing it with combinations of glucose (25mM), 3-o-methylglucose (25mM) or PJ-34 (1uM) during the following 2 days. After 2 days in low glucose, selected experiments evaluated the acute effects of glucose (25mM), 3-o-methylglucose (25mM), PJ-34 (1uM) or gurmarin (30ug/ml) by spiking stock volumes in the wells and harvesting 6h after. Signaling experiments were performed at day 6 of differentiation after 10min of equilibration in EBSS and subsequent incubation of 2x stocks in EBSS to achieve 10mM sucralose, 10μM PD98059, 10ng/ml EGF or 0.1mM PMA.

#### Oxygen consumption in isolated fibers and primary cultures

Measurement of mitochondrial function (oxygen consumption) in muscle fibers and cells was performed as described^6^. Oxygen consumption in saponin-permealized fibers was performed using Oxygraph 2K (Oroboros, Austria). Maximal respiration supported by electron flux was measured with the addition of ADP (500 mM) after stabilization on saturating pyruvate-malate (2M). Steady-state O2 flux for was determined and normalized to fiber dry weight using Datlab 6 software (Oroboros). Primary cells were seeded at 6000cells/well into XF96 culture plates, differentiated, and assayed in XF assay medium containing 4.5g/L glucose and 0.11g/L pyruvate. Basic respiration was assayed by sequentially injecting 0.8ug/ml oligomycin, 1μM FCCP and 2μM antimycin.

#### RNA isolation, gene expression and analysis

Frozen tissue samples were homogenized with 1mL of TRIzol (Ambion), 3 2.8mm glass beads (Omni International, 19-646-3), and 1 0.05mL scoop of 0.05mm glass beads (Next Advance, GB05-RNA) at 0°C × 6800RPM × 20sec x10sec pause × 9 cycles. Homogenization was repeated 5 times. Samples were separated from beads and mixed with 200 μL of chloroform in 1.5mL Eppendorf tubes. Samples were centrifuged at 12,000g × 4°C × 15min. Clear phase was separated, and ethanol (200 proof) was added 1:1 with clear phase. A Zymo Research Direct-zol RNA Microprep kit (catalog no. R2062) was used for purification. RNA concentration was measured using nanodrop. 1μg RNA was reverse transcribed using the High-Capacity cDNA Reverse Transcription kit (Applied Biosystems). cDNA was brought to 100μL and 2μL of sample per well was used in real time PCR using SYBR chemistry (BioRad). The cycle thresholds were compared to the 18s housekeeping gene and gene expression was expressed in arbitrary units as 2^−ΔCt^.

#### Ribotag RNA isolation and analysis

Muscle samples were harvested, washed in PBS with 100 μg/ml cycloheximide, and frozen in liquid nitrogen. Frozen samples were pulverized and homogenized in 1mL of cold polysome buffer (20 mM Tris pH 7.4, 10 mM MgCl, 200 mM KCl, 2 mM DTT, 1% Triton X-100, 100 μg/ml CHX) using 2mL Dounce homogenizer. Homogenates were transfered to a 1.5ml RNase-free tube and centrifuged at 17,000xg, 4°C, for 10 minutes. The supernatant was brought to a fresh 1.5mL tube and 100μL of sample was saved as input. The remaining supernatant was incubated with 4μL of anti-hemagglutinin antibody (catalog number) for 3–6 hours at 4°C with constant rotation. 50μL of AG Magnetic Beads (catalog number) were washed 5 times with polysome buffer, re-suspended into 50μL of polysome buffer, and added to the samples. Following overnight rotation, the samples were placed on a magnetic rack and washed 5x with 500μL of high salt buffer (20 mM Tris pH 7.4, 10 mM MgCl, 300 mM KCl, 2 mM DTT, 1% Triton X-100) before elution with 300μL of TRIzol. The supernatant was mixed with an equal volume of ethanol and RNA was immediately extracted using a purification kit (Zymo Micro). Reverse transcription and real time PCR were performed thereafter with our standard protocol. Gene enrichment was calculated as the ration between elution and input expression. Associations with cell markers were evaluated through principal component analysis of eluate gene expressions after normalization with 18s cDNA.

#### Muscle fiber CSA, fiber number and nuclei number

Muscles were dissected, fixed with 1% PFA in PBS for 1h at RT, and cryo-protected in 30% sucrose overnight. Muscles were then frozen in OCT using nitrogen-cold methyl-butane, kept on dry ice and cut in 12μm sections. Slides were then brought to RT, washed with PBS 0.05% tween, washed again with PBS, and incubated overnight in a humid chamber at 4°C with primary antibodies. Slides were then brought to RT, washed with PBS 0.05% tween, washed again with PBS, and incubated for 1h in a humid chamber at RT with secondary antibodies. Slides were then washed with PBS 0.05% tween, incubated with DAPI, washed with PBS, mounted with IF mounting medium (Southern Biotech Fluoromount G 0100–01) and placed in a cooled to RT foil-covered dessicating chamber with EMD-Millipore-Sigma DX0017 beads. Images were captured with Cytation 1 using Gen5 software (BioTek). Images were then stitched and analyzed with a laminin primary mask to define the fibers. Fiber type and associated nuclei were determined by the image channels.

#### Immunohistochemistry and tissue fluorescence

For SDH, tissues were frozen in OCT, then sectioned and stained within 5 days of freezing. Slides were incubated in SDH solution (48mM succinate, 1mM 1-methoxyphenzine methosulphate, 1.5mM Nitroblue tetrazolium in PBS) for 10 minutes and then dipped into ddH2O to stop the reaction. Slides were washed in 1X PBS and blocked for 45 minutes. Laminin was applied as primary antibody and used thereafter as primary mask. mKO slides were imaged using Cytation 1 and Gen5 software (BioTek). OB mice slides were imaged in the SBP core using Aperio Scanscope (Leica) software. For TdTomato tissue fluorescence, muscles were cryo-protected in 30% sucrose overnight, then frozen in OCT, sectioned, mounted (Prolong Gold Antifade Mountant), and imaged using Zeiss LSM 900 confocal with a 581nm filter and Zen 3.0 Zeiss Confocal software.

#### Electron Microscopy

Transmission electron microscopy imaging of muscle samples was performed by the OSU imaging core. Soleus samples were specially chosen for its fast fixation, thus preserving mitochondrial ultrastructure. Samples were dissected and fixed in 2.5% glutaraldehyde in 0.1 M phosphate buffer. Samples were postfixed with 1% osmium tetroxide and then *en bloc* stained with 1% aqueous uranyl acetate, dehydrated in a graded series of ethanol, and embedded in Eponate 12 epoxy resin (Ted Pella Inc., Redding, CA). Ultrathin sections were cut with a Leica EM UC7 ultramicrotome (Leica microsystems Inc., Deerfield, IL) and collected on copper grids. Images were acquired with an FEI Technai G2 Spirit transmission electron microscope (Thermo Fisher Scientific, Waltham, MA) operating at 80kV, and a Macrofire (Optronics, Inc., Chelmsford, MA) digital camera and AMT image capture software. For random sampling during imaging, at least 3 myofibers per subject were identified and then each was imaged in at least 4 random locations. The process was repeated at 11500x and 34000x.

#### NAD cyclic assay

NAD was determined by a cycling assay based on alcohol dehydrogenase after the acidic destruction of NADH^7^. 500μL of ice-cold 0.6M perchloric acid was added to pelleted cells or to 23mg of lyophilized tissue placed in 500μL skirted tubes with 5mg of glass beads. Samples were homogenized at 0°C in a Precellys homogenizer with 3 cycles of shaking at 7200RPM × 20sec and 15sec pause, repeated 4 times. The homogenates were centrifuged at 12000g during 20min and 4°C, and 300μL of acidic extract was saved in a new ice-cold 1.5ml tube and the acidic pellet was saved for protein determination. Samples were then diluted 1:10 (cells) or 1:100 (tissues) with 100mM PBS right before starting the assay. The assay was performed in triplicate in black opaque 96 well plates containing 5μL of sample and standards and 95μL of freshly-made cycling assay (100mM PBS, 0.1% BSA, 10mM NAM, 2% ethanol, 10μM FMN, 20μM resazurin, 100μg/μL ADH, 10ug/ml diaphorase) and 530/590 fluorescence was recorded for 30min. NAD from muscle samples was normalized by mg of dry tissue. For cell determinations, the total protein per well was used to normalize NAD. The acidic cell pellet was centrifuged again at 12000g during 20min and 4°C and any remaining supernatant was discarded. The pellet was then neutralized with 50μL 1M NaOH, and homogenated at 7200RPM × 20sec three times or until solubilization. The basic homogenate was then spun and neutralized with 250μL 100mM PBS before measuring protein with a BCA kit.

#### Muscle metabolomics

Targeted, quantitative metabolomics of pyridines was performed from lyophilized gastrocnemius muscle as previously described^8^. Briefly, oxidized nucleotides were extracted in 0.5M perchloric acid, stabilized with 1M ammonium formate and separated by liquid chromatography on a Hypercarb column (Thermo) with a mobile phase of 10 mM ammonium acetate pH 9.5 and a gradient of acetonitrile. Nucleotides were quantified by single reaction monitoring using a Quantiva triple quadrupole (Thermo) operating in positive ion mode and an electrospray ionization capillary voltage of 3500 V.

#### Protein extraction

4–6mg of lyophilized powdered tissue was weighed in 2ml Sarstedt tube and protein was extracted with 200μL of RIPA buffer supplemented with protease and phosphatase inhibitors (Roche). Samples were placed 10min on ice and then homogeneized with 3 cycles of shaking at 7200RPM × 20sec and 15sec pause in a Precellys. After spinning at 12000g during 30min, supernatants were placed in new tubes and protein was measured immediately with a BCA kit. Samples were then brought to 2mg/ml with supplemented RIPA buffer and 6x reducing buffer and stored at −20°C if not used immediately.

#### Immunoblotting

Samples were heated for 10 minutes at either 65 °C (samples probed for parylation) or 85 °C and 10–30μg of protein per lane was loaded into standard 7–10% reducing acrylamide gels for electrophoresis. Protein transfer onto PVDF (for mitochondrial proteins) or nitrocellulose membrane was performed in Towbin buffer at 100 V for 90 minutes inside a cold 4°C room. After the transfer, blots were Ponceau-stained, cut into pieces to match the molecular weight of a target and a housekeeping protein, destained, and blocked with 5% dehydrated milk in TBST buffer. Blots were then probed overnight at 4°C with corresponding primary antibodies and continuous rocking. After 3 washes in TBST, blots were incubated for 1h with secondary antibodies, washed again and visualized with SuperSignal reagent in a Bio-Rad ChemiDoc imaging system. Original images are shown at “Resource Data images” file.

#### Nuclear isolation

Lyophilized, pulverized muscle powder (12–15 mg) was resuspended in 300 μL PBS and homogenized via dounce homogenizer. Homogenate was centrifuged at 1900 × g, 4 °C for 15 minutes, and supernatant was isolated as cytosolic fraction. The pellet was resuspended in 150 μL CER I buffer and incubated 10 min on ice. Buffer CER II (8.25 μL) was added to each sample, incubated 1 min on ice, then centrifuged at 16,000 × g, 4 °C for 5 min. Supernatant was collected and added to the cytosolic fraction. Pellet was resuspended in 75 μL NER and incubated on ice for 40 min, with vortexing every 10 min. Samples were centrifuged at 16,000 × g, 4 °C for 10 minutes. Supernatant was collected as nuclear fraction. Sample protein concentration was determined via BCA assay.

#### Immunoprecipitation

Protein samples (450 μg of each) were diluted to 1 μg/μL in RIPA buffer with protease and phosphatase inhibitors. To pre-clear samples, 50 μL Protein A/G-conjugated agarose beads were added to each sample and incubated for 60 min. at 4 C while rotating. Beads and supernatant were separated magnetically, and 50 μL of supernatant was isolated as “input” sample. Remaining supernatant was incubated with 4 μL PARP antibody overnight at 4 °C with rotation. Protein A/G-conjugated agarose beads (50 μL) were added to each sample and incubated at 4 °C for 2 hours. Beads and supernatant were separated magnetically, and supernatant was discarded. Beads were washed 5X in 500 μL cold RIPA with inhibitors. SDS 6X reducing buffer was added (15 μL) and samples were incubated at 65 °C for 8 minutes to elute protein, after which 15 μL RIPA with inhibitors was added and protein samples were magnetically separated from beads.

### Human studies

#### Study design and participant characteristics

The study was a randomized controlled trial with a parallel group design between conducted between 2012 and 2017 at the University of Pittsburgh and the AdventHealth Translational Research Institute (AH TRI). Eighty-four physically inactive men and women aged between 60–80 years of age and with obesity were randomized into one of three 6-month interventions with a 1:1:1 allocation ratio; Health education control (CON;); calorie restriction-induced weight loss (WL), and weight loss with exercise (WLEX). Written informed consent was obtained from all participants prior to participation. Randomization was performed electronically using a random allocation sequence designated by the study statistician. A permuted-blocks approach using blocks of random sizes of 4 and/or 8 was used, with groups stratified by gender. The study coordinator was responsible for participant enrollment and group assignment. Outcome assessors were blinded to group assignment. Sixty-one participants received allocated intervention. The study protocol was approved by both University of Pittsburgh Research Ethics Board and Institutional Review Board of AdventHealth. The study was also registered on ClinicalTrials.gov (NCT02230839).

Participants were enrolled in the study if they met the following screening criteria: BMI > 30 kg/m2; stable weight over the last 6 months; physically inactive (≤1 continuous exercise session/week); non-smoking; resting systolic blood pressure (SBP) < 150 mmHg and diastolic blood pressure (DBP) < 95 mmHg. Exclusion criteria included: clinically significant CVD including history of myocardial infarction within the past year; peripheral vascular disease; hepatic, renal, muscular/neuromuscular, or active hematologic/oncologic disease; presence of bruits in the lower extremities, history of pulmonary emboli; peripheral neuropathy; anemia; substance abuse. Medication exclusions included the following: anticoagulants, glucocorticoids, thiazolidinediones, or insulin.

#### Health education control (CON) intervention

Participants randomized to the CON group received bi-weekly general health education seminars on medication and type 2 diabetes management. However, they were not given specific exercise or dietary education.

#### Calorie restriction-induced weight loss (WL) intervention

The goal of the WL intervention was to lose 10% of baseline body weight. To achieve this goal, we used the Harris-Benedict equation with a correction for physical activity factor, to calculate a reduction of 500–1000 kcal/day based on baseline body weight. This reduction in caloric intake was prescribed along with a low-fat (< 30% of kilocalories from fat) diet. To encourage compliance, participants met individually with the Registered Dietitian and/or designated staff weekly to record body weight, review daily food logs, and receive updated dietary guidelines. To eliminate the confounding effects of acute caloric restriction on insulin sensitivity, participant weights were kept stable during the last two weeks of the intervention.

#### Weight loss and exercise (WLEX) intervention

The WLEX group completed a progressive 6-month exercise training program. The program consisted of exercise on 4–5 days per week, with 45 minutes per session (180 minutes per week) and consisting of mostly walking (outside and on an indoor treadmill) with the option of utilizing stationary cycling, elliptical and rowing machines. Endurance exercise was performed at 50–80% HRreserve. Exercise performed indoors at the training facility was supervised by a trained research assistant, while exercise performed outdoors was not supervised. The WLEX group was also prescribed two, non-consecutive resistance 30 min session exercise sessions per week starting at week 8. The exercises focused on major muscle groups using resistance exercise machines. Nine exercises (2–3 sets of 10–12 repetitions) were performed that alternated between upper and lower limbs, and trunk. The resistance exercises were performed at the highest weight the participant could achieve for the given number of reps (10–12) with proper form. When the participant reached 3×12 reps, the weight was increased, and the number of reps were decreased. Participants also met with the Registered Dietitian and received identical dietary instruction as the WL group.

#### Blood analyses

Glucose and HbA1C were measured by a fasting blood draw and analyzed in the clinical chemistry laboratory at AH TRI using standard assays. Blood was also drawn, centrifuged, and buffy coat was collected and frozen for DNA isolation and genotyping.

#### Cardiorespiratory fitness, muscle strength, and mitochondrial capacity

A cardiopulmonary graded exercise test was conducted by an exercise physiologist on the cycle ergometer using open circuit indirect calorimetry to measure maximal oxygen uptake (VO2peak). Following a standardized warm-up, participants exercised at a moderate intensity and resistance increased gradually until volitional fatigue.

Muscle strength and power were assessed at baseline and 6 months using a pneumatic-driven dynamometer (Biodex 4, Biodex Medical Systems, Inc., NY, USA). Following a one-minute warmup of free pedaling on a cycle ergometer, participants were seated on the Biodex machine with the lateral condyle of the knee lined up with the axis of rotation of the machine arm. Participants performed three tests on each leg at each resistance of 60, 120 and 180 degrees per second with a ~2-minute rest between each adjustment and a 5-minute rest between legs. Muscle average power and torque were calculated as the average of 60-, 120- and 180-degree tests.

*In vivo* muscle mitochondrial function was assessed at rest in the vastus lateralis using phosphorus (^31^P) magnetic resonance spectroscopy on the 3-T magnet as previously described^9^.

#### Body composition

Weight and height were measured pre- and post- intervention and BMI was calculated. Waist circumference was measured using the Gulick II tape measure directly on the skin. Fat mass (FM; kg) and fat-free mass (FFM; kg) were determined by dual-energy X-ray absorptiometry (DXA) using a GE Lunar (GE Healthcare, UK). Skeletal muscle index was calculated as appendicular lean mass normalized by the square height.

#### Genotyping

The Ile191Val TAS1R2 gene polymorphism (rs35874116) was determined by a TaqMan allelic discrimination assay (TaqMan # 4351379, ThermoFisher Scientific). Out of the 61 allocated participants, only 41 had available blood samples and complete pre- and post- outcome data to conduct a retrospective genotyping (CON, n = 15;WL, n = 12;WL + EX, n = 14).

### Statistics

#### Inferential statistics

Inferential statistics were performed by null hypothesis significance testing at a significance level of 0.05 and assuming normal distributions. Two-tailed tests were systematically employed throughout to infer a difference against the null. One-tailed tests were performed in a small number of preclinical experiments that were specifically designed to test directional outcomes after substantial precedents were accumulated.

#### Preclinical data

Preclinical data was analyzed with GraphPad Prism 9. Planned comparisons between two groups were analyzed by t-tests, while non-orthogonal planned comparisons were performed through Šidák-corrected values after ANOVA. Planned comparisons and post-hoc tests within 2-factor designs were also Šidák corrected after ANOVA, except two western blot experiments run in multiple gels where t-tests were employed. Linear regressions were performed to either test for a significant association between variables or to compare adjusted means between two groups. For the latter, no significant difference between slopes was observed in any case.

#### Clinical data

Clinical data was analyzed with jamovi 2.2.5. Genotyping results were tested for Hardy-Weinberg equilibrium and allele distribution against a standard population using Chi square tests. For the retrospective evaluation of the genotype effects, we considered a factorial design (intervention group and genotype) which was corrected for sex, age and diabetic status to limit any effect of confounding factors (GAMLj linear regression module). Baseline values were tested for genotype differences (Welch) and random allocation (ANOVA and Tukey-corrected post-hocs).

Delta values were used to analyze the intervention effect as recommended for retrospective studies^10^. The main intervention effect (independent of genotype) on each measured outcome was tested through ANOVA and Tukey-corrected post-hoc. Genotype differences within the WLEX group were also tested within the regression module as a single independent contrast. The possibility that the two genotype subpopulations would respond differently to health education control (CON) was discarded by Welch tests (Extended Data Table 4). The intervention effects for each genotype subpopulation were then analyzed as independent Welch-corrected tests against the pooled control group to overcome an imbalance of the retrospective factorial design (i.e., CON: Ile n=10; Val n=5).

The primary goal of the retrospective analysis was to evaluate whether Ile/Ile and Val/_ participants had different cumulative responses to the exercise intervention. We evaluated this comprehensive response through the analysis of composite indexes. Z-scores were first calculated in Microsoft Excel 2016 by centering and scaling all delta adjusted values to the pooled control group. The main intervention direction of each variable (+1 or −1) was then employed to transform the z-score values before averaging them in composite response indexes. Genotype differences to the intervention were then analyzed by a Welch-corrected contrast, and its size effect was calculated as a Cohen’s d. Genotype differences against the CON group were also analyzed by independent Welch tests to evaluate if the intervention was effective for each genotype.

## Extended Data

**Extended Data Table 1:** Effects of diet-induced weight loss with or without exercie training in older sedentary obese participants

					*posthoc, P*			
	CON n=15	WL n=12	WLEX n=14	*Main, P*	CON vs. WL	CON vs. WLEX		WL vs. WLEX
**Body Composition**								
ΔBMI (Kg/m^2^)	−0.32 ±0.34	−1.82 ±0.54	−3.43 ±0.49	**0.001**	0.097	**0.001**		0.128
Δ Body mass (Kg)	−1.22 ±0.87	−5.84 ±1.47	−9.65 ±1.26	**0.001**	0.056	**0.001**		0.183
Δ Fat mass (%)	−0.25 ±0.37	−1.66 ±0.75	−4.15 ±0.55	**0.000**	0.236	**0.000**		**0.025**
Δ Lean mass (%)	0.32 ±0.36	1.65 ±0.73	4.16 ±0.55	**0.000**	0.282	**0.000**		**0.023**
Δ Arms lean mass (Kg)	−0.15 ± 0.08	−0.38 ±0.10	−0.07±0.17	0.284	0.479	0.919		0.279
Δ Legs lean mass (Kg)	−0.41 ±0.28	−0.34 ±0.33	0.08 ±0.34	0.580	0.997	0.613		0.663
Δ Skeletal muscle index (kg/m^2^)	−0.18 ±0.13	−0.22 ±0.13	0.03 ±0.17	0.446	0.944	0.630		0.440
**Glucose Control**								
Δ Glucose fasting (mg/dL)	5.08 ±6.09	−3.32 ±4.35	−12.71 ±4.90	0.137	0.654	0.116		0.489
Δ Insulin fasting (mlU/mL)	−4.20 ±1.11	−7.32 ±2.05	−7.65 ±1.77	0.510	0.770	0.492		0.890
Δ HOMA-IR	−0.60 ±0.46	−2.23 ± 0.48	−2.32 ±0.45	0.107	0.198	0.148		0.971
Δ HbA1c (%)	−0.21 ±0.16	−0.46 ± 0.08	−0.78 ± 0.21	0.152	0.002	0.137		0.400
**Aerobic Capacity**								
Δ VO_2 peak_ (L/min)	−0.02 ±0.04	−0.07 ±0.03	0.11 ±0.05	0.057	0.S26	0.161		0.061
Δ VO_2 peak_ (mL/Kg_FFM_/min)	0.10 ±0.80	−1.12 ±0.71	2.16 ±0.83	0.065	0.747	0.214		0.063
Δ RER _peak_	−0.03 ± 0.02	0.01 ±0.02	0.04 ±0.01	**0.019**	0.236	**0.014**		0.454
Δ Ergometer work _peak_ (W)	6.30 ±5.17	−8.53 ±5.64	29.61 ±5.19	**0.001**	0.314	**0.020**		**0.001**
**Mitochondrial Capacity**								
Δ k; PCr recovery rate constant (1/sec*10^3^)	1.19 ±1.88	−0.81 ±2.19	10.18 ±2.86	0.063	0.906	0.102	0.072	
Δ Q_max_; ATP rate (mM/sec)	0.04 ±0.05	−0.04 ±0.05	0.19 ±0.07	0.137	0.762	0.27S	0.126	
**Muscle Fitness**								
Δ Muscle ave. torque _peak_ (Nm)	−2.97 ±1.69	−5.07 ±1.29	0.99 ± 1.78	0.119	0.744	0.340	0.110	
Δ Muscle ave. power _peak_ (J)	−8.62 ±2.36	−10.90 ± 2.10	−1.48 ± 2.47	0.075	0.871	0.181	0.083	
Covariates								
Age (y)	69.53 ± 1.16	68.92 ±1.29	67.36 ±0.87					
Sex (F;M)	12; 3	10; 2	9; 5					
Diabetes	5	3	5					

All values are mean ± SEM of post-pre treatment change (Δ) after adjustment for sex, age,and diabetes status. P values for treatment main effect correspond to the group factor of the two-way ANCOVA. Between-group comparisons were obtained with Tukey’s post-hoc tests, p values <0.05 are shown in bold. CON, health education only control; WL, diet-induced weight loss; WLEX, WL with exercise training; BMI, body mass index; HOMA-IR, Homeostatic Model Assessment for Insulin Resistance; HbA1c, glycated hemoglobin Ale; VO_2_, O_2_ consumption during peak exercise; RER, respiratory exchange ratio during peak exercise; ^31^P-MRS, phosphorus magnetic resonance spectroscopy; PCr, phosphocreatine; OXPHOS, oxidative phosphorylation; ETS, electron transport system; L, leak respiration.

**Extended Data Table 2:** Effect of the TASlR2-llel91Val genotype on baseline (pre treatment) measurements across groups

	Combined			CON		WL		WLEX		
	lle/lle n=22	Val/_ n=19	Main, *P*	lle/lle n=10	Val/_ n=5	lle/lle n=5	Val/_ n=7	lle/lle n=7	Val/_ n=7	Interaction, *p*
**Body Composition**										
BMI (Kg/m^2^)	35.57 ±0.94	38.31 ±1.11	0.068	33.97 ±1.64	35.69 ±2.19	35.69 ± 2.06	35.41 ± 1.91	36.36 ±1.85	39.93 ± 1.83	0.581
Body mass (Kg)	98.52 ±3.64	102.19 ±2.94	0.438	94.36 ±5.66	102.37 ± 7.55	101.72 ± 7.10	101.10 ± 6.58	103.44 ±6.36	107.62 ±6.30	0.794
Fat mass (%)	45.93 ± 1.38	49.07 ±1.42	0.120	42.22 ± 1.40	41.66 ± 1.95	44.47 ± 1.85	43.66 ± 1.71	45.37 ±1.54	48.06 ± 1.65	0.488
Lean mass (%)	51.22 ±1.31	48.35 ±1.32	0.133	54.71 ±1.32	55.35 ±1.83	52.52 ±1.74	53.41 ±1.61	51.74 ±1.44	49.22 ± 1.55	0.464
Arms lean mass (Kg)	5.95 ±0.37	5.56 ± 0.43	0.506	6.29 ± 0.42	7.44 ± 0.58	6.61 ±0.55	7.19 ±0.51	6.73 ± 0.46	5.74 ± 0.49	0.085
Legs lean mass (Kg)	17.50 ± 0.68	17.27 ±0.66	0.814	17.71 ± 0.85	20.15 ±1.18	17.91 ±1.12	18.60 ±1.04	19.15 ±0.93	17.98 ± 1.00	0.200
Skeletal muscle index (kg/m^2^)	8.48 ± 0.21	8.47 ±0.23	0.971	8.59 ± 0.28	9.58 ± 0.40	8.53 ±0.38	8.86 ±0.35	9.02 ±0.31	8.61 ±0.34	0.125
**Glucose Control**										
Glucose fasting (mg/dL)	104.41 ±4.26	106.89 ±4.90	0.704	107.38 ±6.96	111.13 ±10.03	117.02 ± 9.51	111.44 ±8.74	104.28 ±7.89	120.26 ±8.47	0.438
Insulin fasting (mlU/mL)	14.75 ±2.24	16.35 ±2.38	0.627	16.53 ±3.34	16.50 ±4.67	11.75 ± 5.09	22.70 ±4.18	17.06 ±4.89	19.90 ±4.72	0.405
HOMA-IR	3.98 ±0.68	4.23 ±0.68	0.799	4.38 ±0.95	4.09 ±1.33	3.28 ±1.45	6.18 ±1.19	4.85 ± 1.40	5.34 ±1.35	0.387
HbAlc (%)	6.01 ±0.15	6.28 ± 0.22	0.314	6.14 ± 0.28	6.47 ±0.40	5.98 ±0.38	6.18 ±0.35	6.28 ± 0.31	6.89 ±0.33	0.808
**Aerobic Capacity**										
VO_2 peak_ (L/min)	1.58 ±0.11	1.45 ± 0.08	0.365	1.85 ± 0.10	1.76 ±0.13	1.33 ±0.14	1.72 ±0.11	1.68 ± 0.10	1.64 ±0.11	0.083
VO_2 peak_ peak (mL/Kg_FFM_/min)	28.98 ±1.45	27.70 ±0.84	0.451	33.43 ± 1.47 #	29.23 ±1.94	22.51 ± 2.06 #	29.49 ±1.70	29.32 ±1.53	29.13 ± 1.64	**0.010**
RER _peak_	1.11 ± 0.02	1.08 ± 0.02	0.332	1.16 ±0.04	1.09 ± 0.05	1.02 ±0.05	1.06 ± 0.04	1.09 ± 0.04	1.08 ± 0.04	0.474
Ergometer work _peak_ (W)	98.68 ±9.06	82.89 ±6.91	0.175	116.29 ±9.89	107.61 ±13.08	78.86 ±13.90	95.47 ±11.43	106.72 ±10.29	95.64 ±11.06	0.416
**Mitochondrial Capacity**										
k; PCr recovery rate constant (l/sec*10^3^)	18.56 ±1.27	20.12 ± 1.62	0.454	17.78 ± 2.17	23.49 ± 3.02	20.95 ±3.31	19.39 ±3.37	19.62 ±2.94	21.76 ± 2.73	0.432
Q_max_; ATP rate (mM/sec)	0.46 ± 0.04	0.50 ± 0.03	0.462	0.45 ± 0.06	0.59 ± 0.08	0.47 ±0.09	0.52 ±0.09	0.51 ± 0.08	0.53 ± 0.07	0.670
**Muscle Fitness**										
Muscle ave. torque _peak_ (Nm)	89.32 ±5.79	76.65 ±4.71	0.098	102.47 ±6.58	100.77 ±8.68	87.28 ±7.33	93.28 ±7.58	103.02 ±6.85	83.34 ±7.31	0.184
Muscle ave. power _peak_ (J)	106.18 ±7.22	89.41 ± 6.35	0.089	126.55 ±8.18	118.01 ± 10.78	103.14 ± 9.10	112.70 ± 9.42	121.07 ±8.51	99.08 ± 9.08	0.196
**Covariates**										
Age (y)	68.59 ± 0.70	68.63 ± 1.14		68.50 ±1.37	71.60 ±2.04	69.20 ± 1.24	68.71 ±2.11	68.29 ±0.81	66.43 ± 1.53	
Sex (F;M)	15; 7	16;3		8;2	4;1	4;1	6;1	3;4	6;1	
Diabetes	8	5		4	1	2	1	2	3	

All values are mean ± SEM after adjustment for sex, age,and diabetes status. P values for the genotype main effect were obtained using Welch-corrected tests on the adjusted values. P values for the allocation correspond to the group x genotype interaction of the 2-way ANCOVA. p values <0.05 are shown in bold. Significant posthoc comparisons (p<0.05; Tukey) between groups are shown using the symbol#. CON, health education only control; WL, diet-induced weight loss; WLEX, WL with exercise training; BMI, body mass index; HOMA-IR, Homeostatic Model Assessment for Insulin Resistance; HbAlc, glycated hemoglobin Ale; VO_z_, 0_2_ consumption during peak exercise; RER, respiratory exchange ratio during peak exercise; ^31^P-MRS, phosphorus magnetic resonance spectroscopy; PCr, phosphocreatine; OXPHOS, oxidative phosphorylation; ETS, electron transport system; L, leak respiration.

**Extended Data Table 3:** Allele distribution of participants

	Allele Frequency	χ^2^	P
**Hardy Weinberg**			
**Ile/Ile**	22 (54%)		
**Ile/Val**	16 (39%)	<0.001	>0.999
**Val/Val**	3 (7%)		
***Allele distribution***			
*Recorded (n=216414)			
**Ile**	68%	0.438	0.601
**Val**	32%		
Observed (n=41)			
**Ile**	73%		
**Val**	27%		

P values for Hardy-Weinberg equilibrium and allele distribution were obtained using chi square tests.

* L. Phan, Y. Jin, H. Zhang, W. Qiang, E. Shekhtman, D. Shao, D. Revoe, R. Villamarin, E. Ivanchenko, M. Kimura, Z. Y. Wang, L. Hao, N. Sharopova, M. Bihan, A. Sturcke, M. Lee, N. Popova, W. Wu, C. Bastiani, M. Ward, J. B. Holmes, V. Lyoshin, K. Kaur, E. Moyer, M. Feolo, and B. L. Kattman. “ALFA: Al lele FrequencyAggregator.” National Centerfor Biotechnology Information, U.S. National Library of Medicine, 10 Mar. 2020,

**Extended Data Table 4:** Effects of the TASlR2-llel91Val genotype on the responses to treatment

	CON			CON	WL				WLEX			
	lle/lle	Val/_	*P*	lle/lle+ Val/_	lle/lle	*P*	Val/_	*P*	lle/lle	*P*	Val/_	*P*
**Body Composition**												
Δ BMI (Kg/m^2^)	−0.29 ± 0.49	−0.36 ±0.52	*0.919*	−0.32 ±0.34	−2.33 ±0.96	*0.104*	−1.45 ±0.65	*0.159*	−3.05 ±0.94	***0.032***	−3.75 ±0.47	***0.000***
Δ Body mass (Kg)	−1.0311.25	−1.52 ±1.21	*0.783*	−1.22 ±0.87	−6.63 ± 2.40	*0.086*	−5.28 ±1.99	*0.097*	−8.96 ±2.56	***0.027***	−10.23 ± 1.06	***0.000***
Δ Fat mass (%)	−0.13 + 0.45	−0.45 ± 0.70	*0.709*	−0.25 ±0.37	−2.18 ±1.83	*0.354*	−1.29 ±0.32	***0.047***	−3.28 ±0.82	***0.009***	−5.02 ±0.63	***0.000***
Δ Lean mass (%)	0.19 ± 0.41	0.56 ±0.73	*0.671*	0.32 ±0.36	2.09 ± 1.79	*0.384*	1.34 ±0.33	***0.050***	3.48 ±0.87	***0.010***	4.84 ±0.62	***0.000***
Δ Arms lean mass (Kg)	−0.17 ±0.11	−0.13 ±0.10	*0.774*	−0.15 ±0.08	−0.32 ±0.15	*0.361*	−0.42 ±0.14	*0.133*	−0.34 ±0.24	*0.480*	0.20 ± 0.21 *	*0.158*
Δ Legs lean mass (Kg)	−0.52 ±0.27	−0.23 ±0.67	*0.702*	−0.41 ±0.28	−0.34 ± 0.48	*0.895*	−0.33 ±0.47	*0.892*	−0.68 ±0.37	*0.581*	0.84 ±0.43*	***0.034***
Δ Skeletal muscle index (kg/m^2^)	−0.24 ±0.14	−0.08 ± 0.27	*0.630*	−0.18 ±0.13	−0.25 ± 0.21	*0.793*	−0.21 ± 0.19	*0.912*	−0.35 ±0.17	*0.451*	0.41 ± 0.21 *	***0,033***
**Glucose Control**												
Δ Glucose fasting (mg/dL)	8.37 ±8.30	−1.51 ±7.93	*0.407*	5.08 ± 6.09	−4.63 ±10.16	*0.439*	−2.39 ±3.13	*0.289*	−0.17 ±3.05	*0.451*	−25.26 ±6.50*	***0.004***
Δ Insulin fasting (mlU/mL)	−3.55 ± 1.44	−5.49 ± 1.75	*0.420*	−4.20 ±1.11	−3.00 ±2.64	*0.706*	−9.48 ± 2.42	*0.087*	−6.29 ±3.21	*0.574*	−8.74 ±2.11	*0.104*
Δ HOMA-IR	−0.33 ±0.64	−1.15 ±0.51	*0.341*	−0.60 ±0.46	−1.56 ±0.56	*0.240*	−2.56 ±0.66	*0.035*	−1.65 ±0.90	*0.353*	−2.86 ±0.27	***0.001***
Δ HbA1c *(%)*	−0.02 ±0.15	−0.60 ±0.34	*0.174*	−0.21 ±0.16	−0.43 ± 0.19	*0.385*	−0.49 ± 0.07	*0.137*	−0.37 ±0.27	*0.613*	−1.18 ±0.26*	***0.010***
**Aerobic Capacity**												
Δ VO_2 peak_ (L/min)	−0.02 ± 0.06	−0.01 ± 0.04	*0.813*	−0.02 ±0.04	0.00 ± 0.03	*0.699*	−0.12 ±0.05	*0.122*	0.05 ± 0.09	*0.516*	0.18 ±0.05	***0.014***
Δ VO_2 peak_ (mk/Kg_FFM_/min)	0.12 ±1.19	0.06 ±1.02	*0.967*	0.10 ±0.80	0.24 ±0.78	*0.901*	−1.89 ±0.93	*0.127*	0.96 ±1.13	*0.543*	3.35 ±1.12	***0,036***
Δ RER _peak_	−0.02 ± 0.02	−0.05 ± 0.03	*0.533*	−0.03 ± 0.02	0.02 ±0.03	*0.114*	−0.01 ± 0.03	*0.487*	0.01 ± 0.01	***0.046***	0.07 ±0.02	***0.001***
Δ Ergometer work _peak_ (W)	7.53 ±6.66	4.33 ±9.10	*0.784*	6.30 ±5.17	−0.12 ±10.03	*0.595*	−13.34 ±6.63	***0.036***	24.21 ± 7.73	*0.080*	35.01 ± 6.87	***0.005***
**Mitochondrial Capacity**												
Δ k; PCr recovery rate constant (1/sec*10^3^)	1.65 ± 1.99	0.44 ±4.03	*0.797*	1.19 ± 1.88	1.03 ± 4.58	*0.977*	−2.19 ±2.20	*0.276*	7.65 ±4.53	*0.256*	12.21 ± 3.84	***0.042***
Δ Q_max_; ATP rate (mM/sec)	0.07 ±0.06	−0.01 ± 0.08	*0.392*	0.04 ± 0.05	0.02 ±0.10	*0.859*	−0.09 ± 0.05	*0.110*	0.15 ±0.12	*0.438*	0.22 ±0.08	*0.110*
**Muscle Fitness**												
Δ Muscle ave. torque _peak_ (Nm)	−3.97 ± 2.49	−1.38 ±1.93	*0.430*	−2.97 ±1.69	−3.06 ±2.41	*0.977*	−6.22 ±1.44	*0.161*	−0.12 ±1.63	*0.240*	2.10 ±3.27	*0.200*
Δ Muscle ave. power _peak_ (J)	−10.36 ±3.41	−5.84 ±2.82	*0.329*	−8.62 ±2.36	−6.70 ±3.86	*0.687*	−13.30 ±2.15	*0.161*	−2.01 ±2.72	*0.087*	−0.94 ±4.36	*0.154*

All values are mean ± SEM of post-pre treatment change (Δ), obtained after adjustments for sex, age, and diabetes status. All individual contrasts were obtained by Welch-corrected tests on the adjusted values. P values under CON represent the within-group genotype effect, while P values under WL and WLEX represent differences against the pooled control group, p values <0.05 are shown in bold. Significant differences between genotypes within the WLEX group are represented by asterisks (*). CON, health education only control; WL, diet-induced weight loss; WLEX, WL with exercise training; BMI, body mass index; HOMA-IR, Homeostatic Model Assessment for Insulin Resistance; HbAlc, glycated hemoglobin Ale; VO_z_, O_z_ consumption during peak exercise; RER, respiratory exchange ratio during peak exercise; ^31^P-MRS, phosphorus magnetic resonance spectroscopy; PCr, phosphocreatine; OXPHOS, oxidative phosphorylation; ETS, electron transport system; L, leak respiration.

## Supplementary Material

1

## Data Availability

The data generated and analyzed for the current study are available from the corresponding author (GAK). The human data that support the findings of this study are not openly available which is stored in a controlled access repository. We will share datasets within the restrictions of institutional review board ethics approvals, upon reasonable request from PMC (Paul.Coen@AdventHealth.com).
