# Supplementary material for: The TAS1R2 sweet taste receptor regulates skeletal muscle mass and fitness
Source: Res Sq. 2023 Feb 9:rs.3.rs-2475555. Preprint. [Version 1] doi: 10.21203/rs.3.rs-2475555/v1 (PMC9934781; doi:10.21203/rs.3.rs-2475555/v1)
Supplement: 1 [file NIHPPRS2475555V1-supplement-1.pdf]

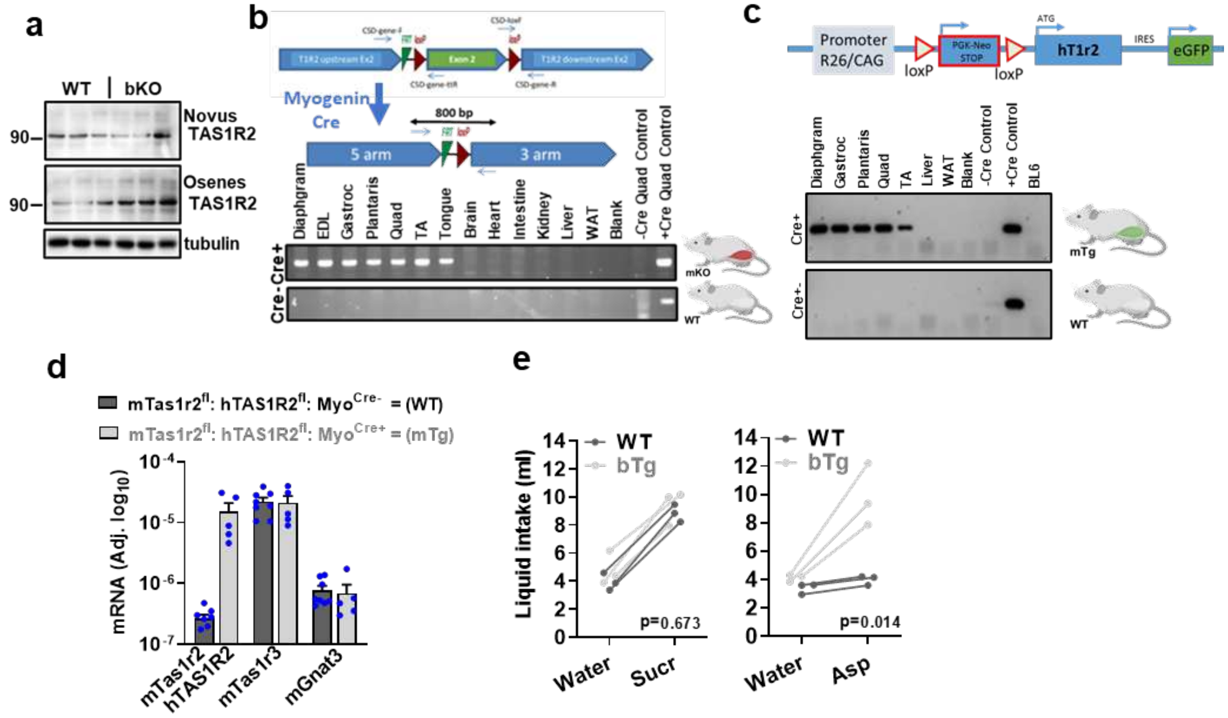

Supplementary Figure 1. Generation of genetic mouse models targeting the Tas1r2 gene. (a) Immunoblotting of mouse TAS1R2 in WT or bKO muscles using two different commercially available antibodies. **(b)** Generation of Myogenin-Cre:Tas1r2-fl/fl mice (mTg) for muscle-specific ablation of TAS1R2. **(c)** Generation of human TAS1R2 transgenic mouse using a R26/CAG promoter fl-stop-fl cassette. **(d)** Expression of endogenous mouse Tas1r2 (mTas1r2) and human TAS1R2 (hT1R2) transgene in skeletal muscles of WT (control) and mTG mice. **(e)** Consumption of sucralose (left) and aspartame (right) sweetened water or water alone in WT and whole-body human TAS1R2 transgenic mice (bTg). Two-way ANOVA, interaction effect.

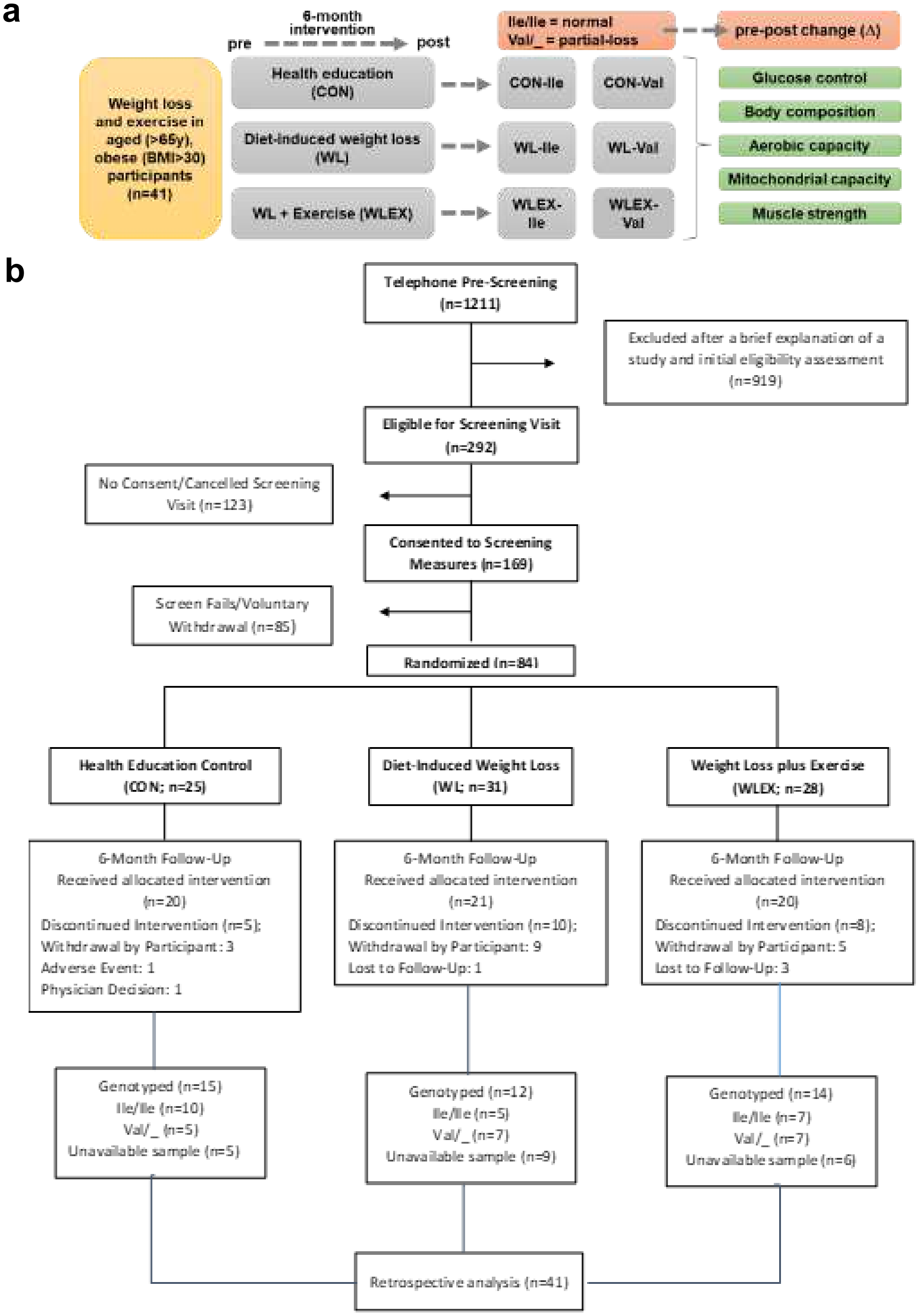

Supplementary Figure 2. Schematic of six-month intervention and patient flow diagram. (a) Effects of TAS1R2-Ile191Val in older obese individuals subjected to 6-months of diet-induced weight loss with exercise training (WLEX), diet-induced weight loss alone (WL), or education control (CON). Participants within each group were retrospectively genotyped and classified as Ile/Ile (i.e., TAS1R2 normal function) or Val/_ (i.e., TAS1R2 partial loss-of-function). The genotype effect was tested in variables associated with glucose control, body composition, aerobic and mitochondrial capacity, and muscle fitness before (pre) and after (post) the interventions. **(b)** Patient flow diagram.
